# Blue Light Regulates Phosphate Deficiency-Dependent Primary Root Growth Inhibition in Arabidopsis

**DOI:** 10.3389/fpls.2019.01803

**Published:** 2020-01-31

**Authors:** Chuan-Ming Yeh, Koichi Kobayashi, Sho Fujii, Hidehiro Fukaki, Nobutaka Mitsuda, Masaru Ohme-Takagi

**Affiliations:** ^1^ Graduate School of Science and Engineering, Saitama University, Saitama, Japan; ^2^ Bioproduction Research Institute, National Institute of Advanced Industrial Science and Technology (AIST), Tsukuba, Japan; ^3^ Institute of Tropical Plant Sciences and Microbiology, College of Bioscience and Biotechnology, National Cheng Kung University, Tainan, Taiwan; ^4^ Graduate School of Arts and Sciences, The University of Tokyo, Tokyo, Japan; ^5^ Graduate School of Science, Kobe University, Kobe, Japan

**Keywords:** BBX32, HY5, light, phosphate deficiency, root architecture, transcription factor

## Abstract

Plants have evolved mechanisms to improve utilization efficiency or acquisition of inorganic phosphate (Pi) in response to Pi deficiency, such as altering root architecture, secreting acid phosphatases, and activating the expression of genes related to Pi uptake and recycling. Although many genes responsive to Pi starvation have been identified, transcription factors that affect tolerance to Pi deficiency have not been well characterized. We show here that the ectopic expression of *B-BOX32* (*BBX32*) and the mutation of *ELONGATED HYPOCOTYL 5* (*HY5*), whose transcriptional activity is negatively regulated by BBX32, resulted in the tolerance to Pi deficiency in Arabidopsis. The primary root lengths of *35S:BBX32* and *hy5* plants were only slightly inhibited under Pi deficient condition and the fresh weights were significantly higher than those of wild type. The Pi deficiency-tolerant root phenotype of *hy5* was similarly observed when grown on the medium without Pi. In addition, a double mutant, *hy5 slr1*, without lateral roots, also showed a long primary root phenotype under phosphate deficiency, indicating that the root phenotype of *hy5* does not result from an increase of external Pi uptake. Moreover, we found that blue light may regulate Pi deficiency-dependent primary root growth inhibition through activating peroxidase gene expression, suggesting the Pi-deficiency tolerant root phenotype of *hy5* may be due to blockage of blue light responses. Altogether, this study points out light quality may play an important role in the regulation of Pi deficiency responses. It may contribute to regulate plant growth under Pi deficiency through proper illumination.

## Introduction

Inorganic phosphate (Pi) is an essential constituent of ATP, nucleic acids, and membrane phospholipids. In addition, it is crucial to various cellular metabolic pathways, including photosynthesis, glycolysis, respiration, signal transduction, and carbohydrate metabolism (Ticconi and Abel, 2004; Péret et al., 2011; Niu, 2013). However, Pi is easily chelated by soil particles or formed insoluble complexes with aluminum or iron at acid pH and with calcium at alkaline pH leading to a low mobility and availability in soils (Wissuwa, 2003; Gaxiola et al., 2011). Therefore, available soil Pi concentrations are often less than the requirement for optimal crop production (Nussaume et al., 2011; Péret et al., 2011; Niu, 2013). Plants have evolved adaptive mechanisms to acquire and recycle Pi in response to Pi deficiency. Alteration of root architecture, such as enhancement of lateral root growth and root hair formation, increases root surface areas for Pi absorption (Ticconi and Abel, 2004; Péret et al., 2011). Induction of high-afﬁnity Pi transporter genes increases uptake of soluble Pi, while activation or secretion of acid phosphatases, ribonucleases, and organic acids enhances scavenging of extracellular Pi from insoluble organic complexes. In addition, the activities of acid phosphatases and ribonucleases also help release Pi from intracellular organic Pi-containing molecules (Raghothama, 2000; Poirier and Bucher, 2002; Nussaume et al., 2011). To improve Pi use efficiency, plants substitute bypass pathways that do not require Pi for metabolic processes requiring Pi (Plaxton and Tran, 2011). Replacing membrane phospholipids with non-P-containing glycolipids also plays an important role in the supply of free Pi during Pi deficiency (Kobayashi et al., 2006).

Many efforts have been made to unravel the molecular mechanisms that regulate Pi starvation responses (PSRs). An array of Pi starvation-induced (PSI) genes have been identified by transcriptome studies (Wu et al., 2003; Misson et al., 2005; Thibaud et al., 2010; Woo et al., 2012) and a series of *hypersensitive to phosphate starvation* (*hps*) mutants have been isolated and characterized (Yeh et al., 2017). Although various plant transcription factors (TFs) affect PSRs, the transcriptional regulation of these processes is not yet well elucidated. *AtPHR1* (*PHOSPHATE STARVATION RESPONSE 1*) is the first Arabidopsis TF gene shown to mediate diverse PSRs (Rubio et al., 2001). Although *AtPHR1* is not Pi starvation-inducible, PHR1 regulates a subset of PSI genes through the miR399-PHO2 (an ubiquitin-conjugating E2 enzyme) signaling pathway (Bari et al., 2006; Chiou et al., 2006). *AtPHR1*, *AtPHL1* (*PHR1-like 1*), and their two rice orthologues, *OsPHR1* and *OsPHR2*, have been identified as having partially redundant functions (Zhou et al., 2008; Bustos et al., 2010; Liu et al., 2010). In addition, several TFs have been identified as negative regulators of PSRs in Arabidopsis. BHLH32, a basic helix-loop-helix TF, negatively regulates anthocyanin accumulation, root hair formation, and induction of the PSI genes (Chen et al., 2007). *AtMYB62* is low-Pi-inducible and mediates its negative effects on PSRs through modulation of gibberellin metabolism (Devaiah et al., 2009). WRKY6 and WRKY42 negatively regulate the expression of *PHOSPHATE1* (*PHO1*), which is responsible for Pi translocation from root to shoot in Arabidopsis (Hamburger et al., 2002; Chen et al., 2009). AtWRKY75 and AtZAT6 have been reported to regulate root development and Pi acquisition, although they may not be specific to PSRs due to their responsiveness to multiple nutrient deficiencies (Devaiah et al., 2007a; Devaiah et al., 2007b). In recent years, several Arabidopsis TF genes, such as *AtERF070*, *APSR1*, *AtMYB2*, and *AL6*, have been shown to be involved in the regulation of root growth and architecture under Pi deficiency (Yeh and Ohme-Takagi, 2015).

Adding Pi fertilizer can improve soil Pi levels; however, the world's Pi rock reserves may be exhausted within 120 years (Gilbert, 2009; Nussaume et al., 2011) and the demand for Pi fertilizers will likely increase to support crop productivity for the growing global population (Nussaume et al., 2011; Péret et al., 2011). In addition, the low solubility of Pi in soils often causes over-application of chemical fertilizers, subsequently leading to potential threats to the environment and the ecosystem (Gaxiola et al., 2011; Péret et al., 2011). Therefore, proper utilization of the remaining Pi reserves is important to reduce Pi resource depletion and environmental threaten. To this end, development of crops with tolerance to Pi deficiency is required, especially if crops can be manipulated to possess higher ability for Pi recycling or Pi utilization efficiency.

In this study, we identified both the chimeric repressor and the ectopic expression of a B-box zinc finger protein (BBX32) gene (35S:BBX32-SRDX and 35S:BBX32) induce Pi deficiency tolerance and the Pi-deficiency tolerance phenotype of *35S:BBX32-SRDX* and *35S:BBX32* may result from the repression of HY5 function. Furthermore, we found that continuous blue light illumination accelerates sensitivity to Pi deficiency in WT and elimination from blue light improves PR growth through reduction of class III peroxidase (PRX) gene expression. Our results indicate that BBX32 and HY5 are involved in the regulation of PSRs, and 35S:BBX32-SRDX and hy5 exhibit root tolerant phenotypes to Pi deficiency due to blockage of blue-light responses.

## Results and Discussion

### Identification of Transcription Factors Responsible for Pi Deficiency Tolerance

To identify transcription factors (TFs) that can be manipulated to allow plants growing well under minimal Pi fertilization, we grew the Arabidopsis Chimeric REpressor gene Silencing Technology (CRES-T) lines in Pi-deficient conditions and screened for plant phenotypes indicative of tolerance to Pi deficiency: larger plant size, longer primary root (PR) length, and lower anthocyanin accumulation than wild type (WT). The CRES-T can convert a transcriptional activator into a strong repressor by fusion with the plant-specific repression domain SRDX (superman repression domain X), leading to dominant repression of the target genes. The resultant transgenic plants exhibit phenotypes similar to those of the knockout mutants of the manipulated TF and its functionally redundant TFs (Hiratsu et al., 2003; Mitsuda and Ohme-Takagi, 2009). In this study, Arabidopsis CRES-T seedlings were grown on 1/2 MS medium with 10 μM Pi (K_2_HPO_4_) to isolate plants that show tolerance to Pi deficiency. A CRES-T line for *BBX32* gene (*35S:BBX32-SRDX*) was isolated and preferentially investigated.

**Figure 1:**
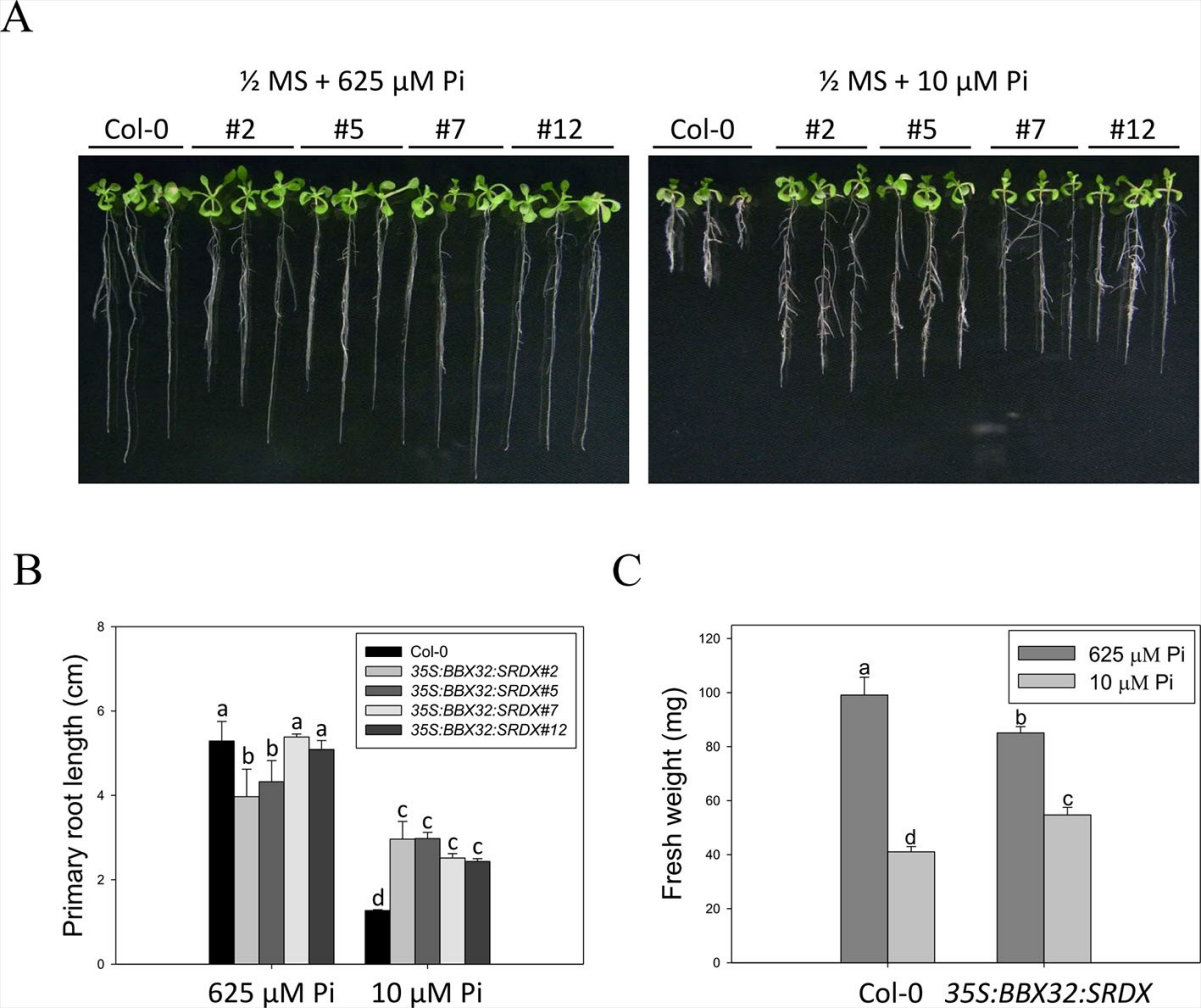
Primary root length and fresh weight of wild-type and *35S:BBX32-SRDX* seedlings in response to Pi treatment. **(A)** Wildtype (Col-0) and *35S:BBX32-SRDX* seedlings (line#2, 5, 7, 12) grown in Pi-sufficient (625 μM) and Pi-deficient (10 μM) conditions. **(B)** Primary root (PR) lengths after growth on vertical plates for 10 days. **(C)** Seedling fresh weights (FWs) after growth on horizontal plates for 16 days. The FW of *35S:BBX32-SRDX* is the average of the four lines used in this study. Data represent the means ± standard error (SE) of four independent experiments. Different letters above the bars indicate statistically significant differences among the means based on Two-way ANOVA (Analysis of Variance) followed by Fisher's LSD (Least Significant Difference) tests (*P < *0.05).

**Figure 2:**
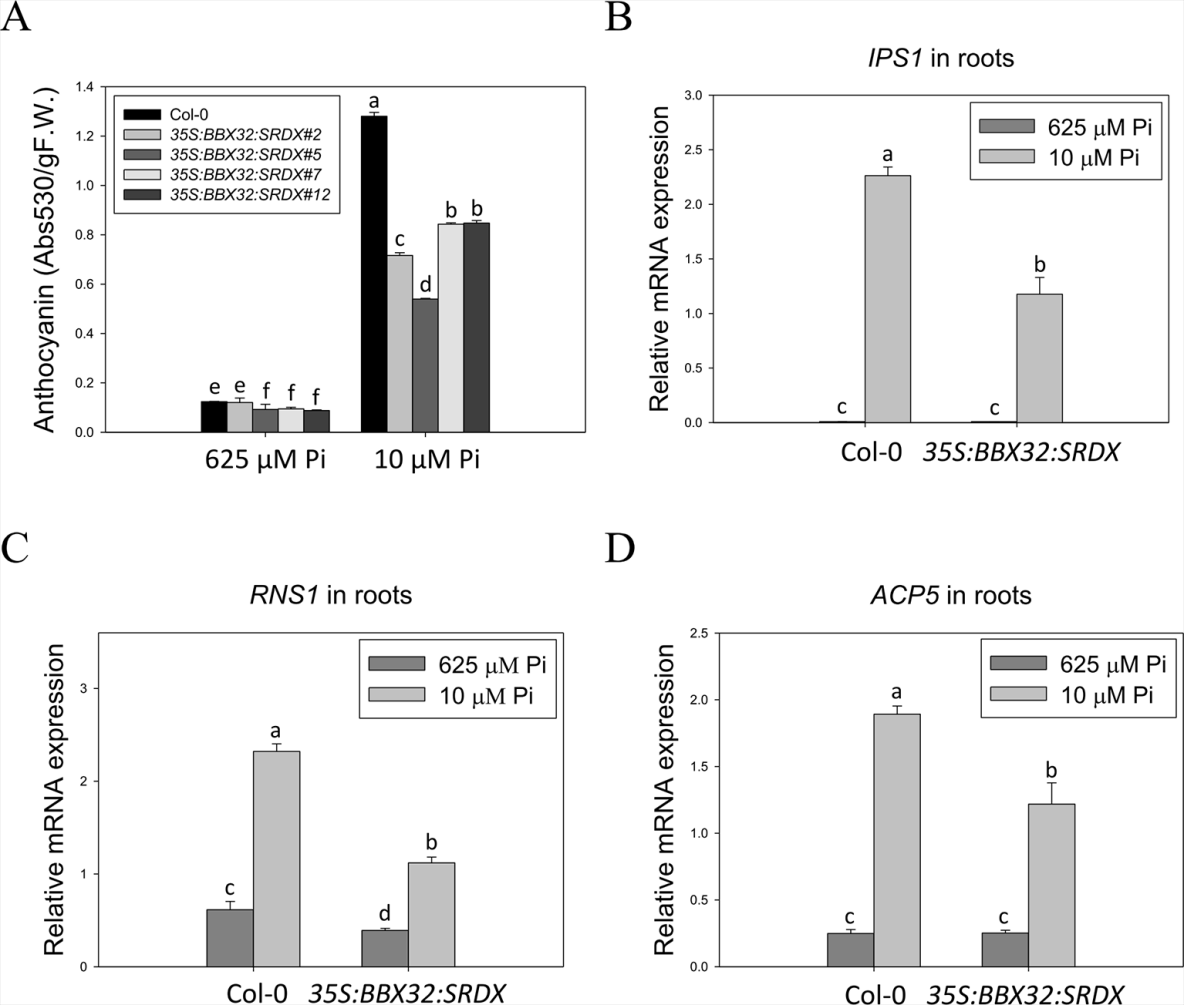
Lower level of anthocyanin content and PSI gene expression in *35S:BBX32-SRDX* plants. Low Pi-induced anthocyanin accumulation in Col-0 and *35S:BBX32-SRDX* **(A)**. Expression level of *IPS1* **(B)**, *RNS1* **(C)**, and *ACP5* **(D)**. Anthocyanin content of 10-day-old seedlings was extracted by 45% methanol and 5% acetic acid. The relative level of anthocyanin was calculated from the absorbance at 530 and 637 nm. RNA extracted from 10-day-old seedlings was subjected to real-time RT-PCR. Data represent means ± standard error (SE) of three independent experiments. Different letters above the bars indicate statistically significant differences among the means based on Two-way ANOVA followed by Fisher's LSD test (*P* < 0.05).

The *35S:BBX32-SRDX* lines and the corresponding WT, Col-0, were grown on 1/2 MS media in the presence of 625 μM Pi (Pi sufficient) or 10 μM Pi (Pi deficient) for 10 days to confirm the tolerant phenotype. The PR growth of both WT and *35S:BBX32-SRDX* was inhibited under Pi deficient condition compared to Pi sufficient condition, while the PR lengths of *35S:BBX32-SRDX* were significantly longer than that of WT (**Figures 1A, B**). In addition, the fresh weight of *35S:BBX32-SRDX* under Pi deficiency was higher compared to the WT (**Figure 1C** and **Supplementary Figure 1**). The Pi-deficiency tolerance of *35S:BBX32-SRDX* was further confirmed by examination of Pi deficiency-responsive anthocyanin accumulation and gene expression. The anthocyanin content was increased in both WT and *35S:BBX32-SRDX* under Pi-deficient conditions; however, the level was significantly lower in *35S:BBX32-SRDX* than in WT (**Figure 2A**). The expression of *INDUCED BY PHOSPHATE STARVATION1* (*IPS1*), *RIBONUCLEASE 1* (*RNS1*), and *ACID PHOSPHATASE TYPE 5* (*ACP5*) was significantly induced by Pi deficiency in the WT, but their transcript levels were much lower in *35S:BBX32-SRDX* than in the WT (**Figures 2B–D**). These results indicate that *35S:BBX32-SRDX* plants appear to suffer less stress in Pi-deficient conditions compared to the WT.

### Phenotype of Overexpression and Knockout Lines of *BBX32*

**Figure 3:**
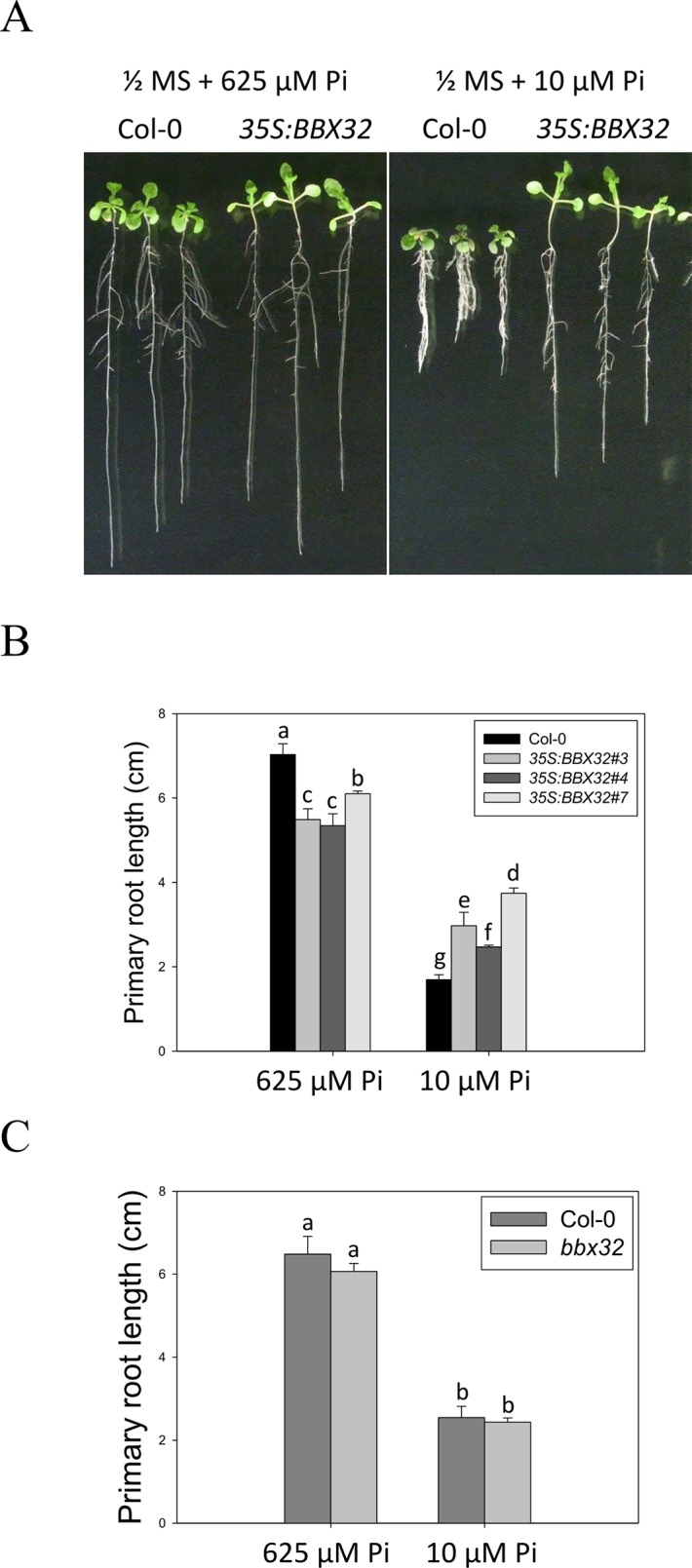
Primary root length of *35S:BBX32* and *bbx32* seedlings in response to Pi treatment. **(A)** Wildtype and *35S:BBX32* seedlings (line#3, 4, 7) grown in Pi-sufficient and Pi-deficient conditions. **(B)** PR lengths of *35S:BBX32* after growth on vertical plates for 10 days. **(C)** PR length of *bbx32* after growth on vertical plates for 10 days. Data represent the means ± standard error (SE) of four independent experiments. Different letters above the bars indicate statistically significant differences among the means based on Two-way ANOVA followed by Fisher's LSD test (*P* < 0.05).

To further examine the role of *BBX32* in response to Pi deficiency, the overexpression lines of *BBX32* (*35S:BBX32*) and *bbx32* knockout plants (SALK_059534) were generated and examined. The *35S:BBX32* plants exhibited a Pi deficiency-tolerant phenotype as *35S:BBX32-SRDX* plants (**Figures 3A, B**), while the inhibition of PR length of *bbx32* under Pi deficiency was similar to that of WT (**Figure 3C**). As shown previously, when a TF is a repressor or inhibitor of transcription, the chimeric repressor and the ectopic expression of a TF exhibit a similar phenotype but show opposite to the knockout line (Matsui et al., 2008; Ikeda et al., 2012). These indicate that BBX32 is likely to play a role in negative regulation in response to Pi deficiency.

### Tolerant Phenotypes of *hy5-215* Mutants Under Pi Deficiency

**Figure 4:**
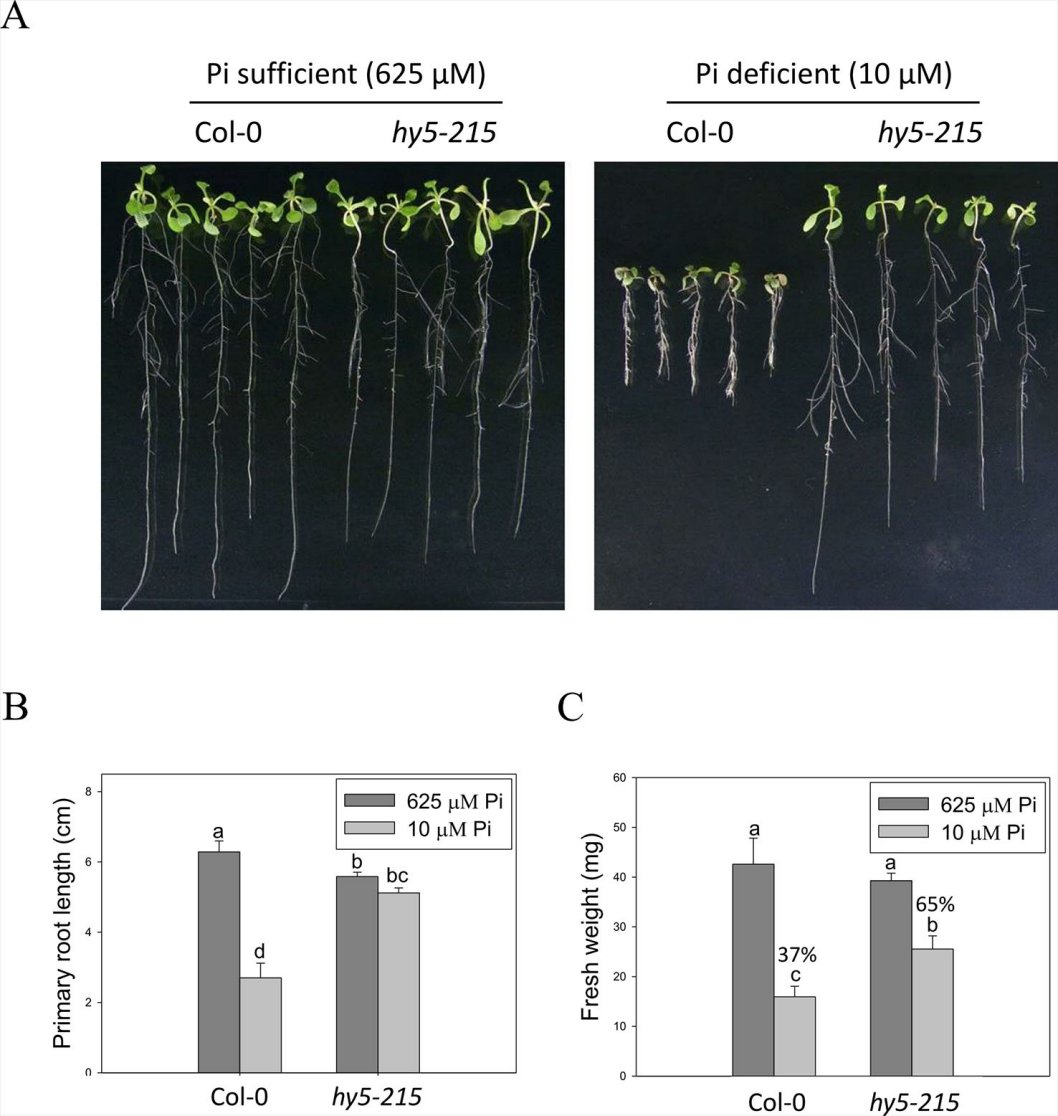
Primary root length and fresh weight of wild-type and mutant seedlings in response to Pi treatment. **(A)** Wildtype and *hy5-215* seedlings grown in Pi-sufficient and Pi-deficient conditions. **(B)** PR lengths after growth on vertical plates for 10 days. **(C)** Seedling FWs after growth on horizontal plates for 14 days. Data represent the means ± standard error (SE) of four independent experiments. Different letters above the bars indicate statistically significant differences among the means based on Two-way ANOVA (Analysis of Variance) followed by Fisher's LSD (Least Significant Difference) tests (*P < *0.05).

**Figure 5:**
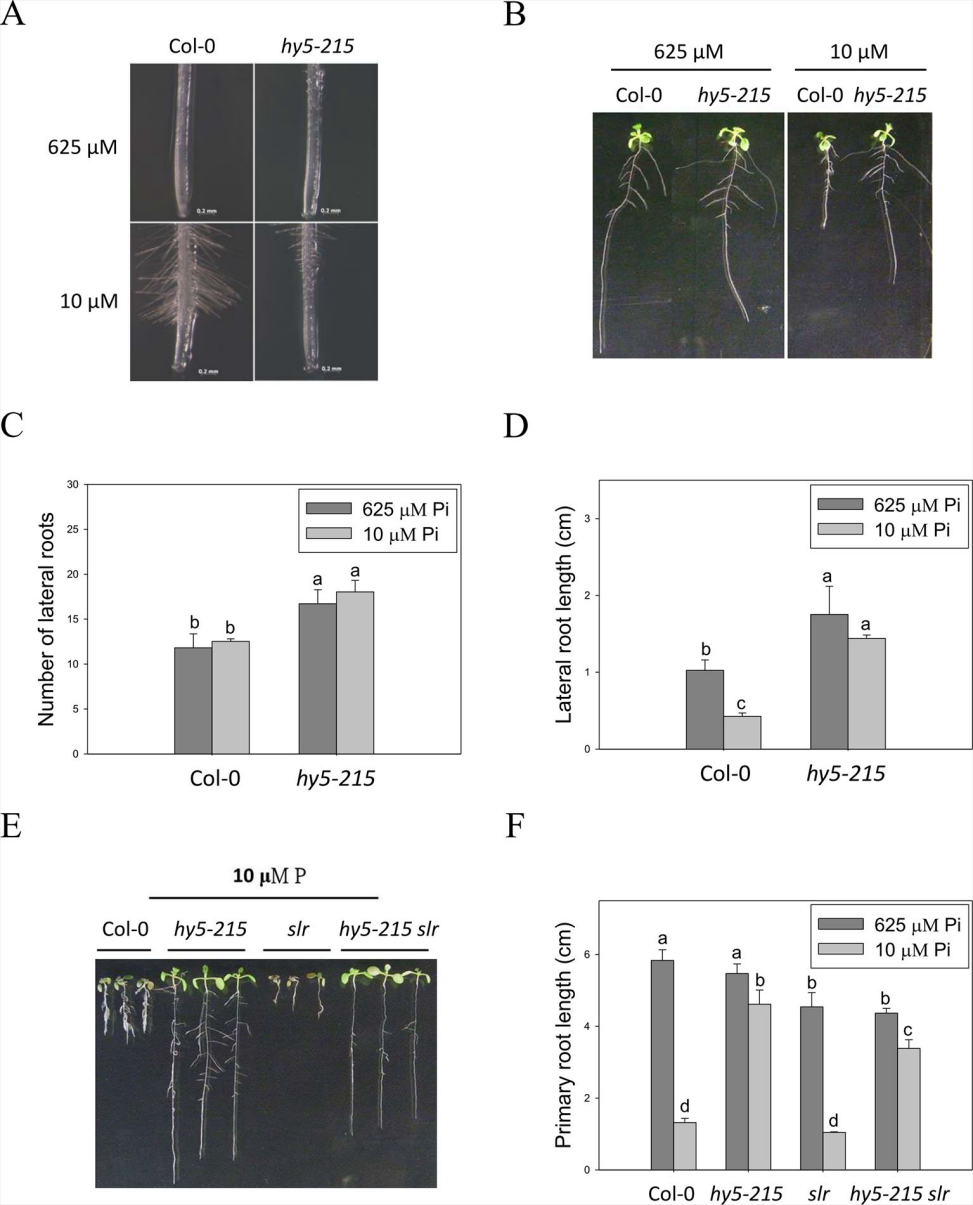
Root hair formation and root architecture of wild-type and mutant seedlings in response to Pi treatment. **(A)** Root hair formation of Col-0 and *hy5-215* after growth of 7 days. **(B)** Root architecture of Col-0 and *hy5-215* after growth of 10 days. **(C)** Increase of LR number in *hy5-215* plants. **(D)** Increase of LR length in *hy5-215* plants. **(E)** The Col-0, *hy5-215*, *slr*, and *hy5-215slr-1* seedlings grown in Pi-sufficient and Pi-deficient conditions. **(F)** PR length in Col-0, *hy5-215*, *slr*, and *hy5-215slr-1*. All the seedlings were grown on 1/2 MS medium with 625 or 10 μM Pi for 7 to 10 days. Data represent means ± SE of four independent experiments. Different letters above the bars indicate statistically significant differences among the means based on Two-way ANOVA followed by Fisher's LSD tests (*P < *0.05).

BBX32 has been reported to modulate light signaling through suppressing activity of ELONGATED HYPOCOTYL 5 (HY5), a bzip TF involved in the regulation of photomorphogenesis. Overexpression of *BBX32* induces hypocotyl elongation in response to multiple wavelengths of light as in *hy5* mutant and represses HY5-regulated gene expression (Holtan et al., 2011). Therefore, we examined whether *hy5* mutant also exhibits Pi deficiency-tolerant phenotype. Here, we used *hy5-215*, generated by ethyl methanesulfonate mutagenesis, for further study (Oyama et al., 1997). The PR lengths of WT were significantly reduced under Pi-deficient conditions when compared with those grown under Pi-sufficient conditions, while only slight inhibition of PR growth was observed in the *hy5-215* mutant between Pi-sufficient and Pi-deficient conditions (**Figures 4A, B**). WT fresh weight declined to 37% under Pi deficient-conditions compared to Pi-sufficient conditions, while *hy5-215* fresh weight declined to 65% under Pi deficient-conditions compared to Pi-sufficient conditions (**Figure 4C** and **Supplementary Figure 2**). We also confirmed the tolerance of *hy5-215* to Pi deficiency by examination of several well-known PSRs including expression of ribonuclease, purple acid phosphatase, and anthocyanin biosynthesis genes (**Supplementary Note** and **Figures 3–5**). These results indicate that the Pi-deficiency tolerant phenotype of *35S:BBX32-SRDX* and *35S:BBX32* plants may indeed result from repression of HY5 function. We, therefore, would use *hy5-215* mutant for further studies to address the tolerance mechanisms.

### Alteration of Root Architecture in *hy5-215* Is Not Responsible to Pi-Deficiency Tolerance

Plant root architecture, the spatial arrangement of a root system, is highly plastic in response to depletion of mineral nutrients. Modiﬁcations of RA through altering the number, length, angle, and diameter of roots or root hairs enable plants to cope with nutrient shortages (Gruber et al., 2013). The “topsoil foraging” strategy is employed to get immobile Pi from the Pi-enriched upper-layer soil under Pi deficiency; in topsoil foraging, plants inhibit PR growth but enhance lateral root (LR) growth and root hair formation, thus increasing the surface area available for Pi uptake (Péret et al., 2011; Sato and Miura, 2011; Niu, 2013). In this study, a great number of root hairs were initiated in the WT under Pi-deficient conditions, whereas *hy5-215* formed fewer and shorter root hairs (**Figure 5A**), suggesting that *hy5-215* may not show as strong of a response to Pi deficiency as WT. Bhosale et al. (2018) reported that Arabidopsis root hair elongation in response to low Pi is regulated by an auxin-dependent pathway mediated by TAA1, AUX1, ARF19, RSL2, and RSL4. We analyzed expression levels of these genes in the roots of WT and *hy5-215* and found no significant difference between WT and mutant (data not shown), suggesting the *hy5-215* phenotype doesn't result from suppression of root hair elongation. It is worth noting that LR numbers and lengths were not enhanced by low-Pi treatment in either WT or *hy5-215*. Instead, LR growth was repressed by our Pi deficiency condition (**Figures 5B–D**). This inconsistency to previous findings may result from different Pi concentrations and experimental conditions used in the different studies. Plants grown at relatively higher levels of Pi (> 1 mM) in Pi-sufficient media form fewer or almost no LRs (Pérez-Torres et al., 2008; Lei et al., 2011). However, Pi-sufficient treatment (625 μM) in this work induces much more LR formation and growth. This is in agreement with the previous reports that used relative lower concentrations for Pi-sufficient treatments (Devaiah et al., 2007a; Pérez-Torres et al., 2008; Devaiah et al., 2009; Lei et al., 2011; Gruber et al., 2013).

Although LR growth was not enhanced by Pi starvation in this study, a root system possessing more and longer LRs was found in *hy5-215* in both Pi-sufficient and Pi-deficient conditions (**Figures 5B–D**). A similar phenotype was found in *35S:BBX32-SRDX* (**Supplementary Figure 6**). To examine whether the increased LR number and lengths contribute to the Pi-deficiency tolerance in *hy5-215*, a double mutant constructed with *hy5-215* and *solitary-root-1* (*slr-1*), a gain-of-function mutant of IAA14 (a repressor of auxin signaling) that does not produce LRs, was examined under Pi deficiency (Fukaki et al., 2002; Kobayashi et al., 2012). The *hy5-215 slr-1* double mutant showed a long-hypocotyl phenotype similar to that of *hy5-215* and a PR lacking LR growth similar to the *slr-1* phenotype. Interestingly, the PR elongation of *hy5-215 slr-1* seedlings was only slightly inhibited by Pi deficiency, although the PR of *hy5-215 slr-1* was shorter than that of *hy5-215* in the respective conditions (**Figures 5E, F**). The results revealed that LR growth is beneficial for growth on Pi-deficient medium, but the change in *hy5-215* root architecture does not appear to be responsible for the observed tolerance to Pi deficiency in *hy5-215*. Auxin signaling was reported to be enhanced in Arabidopsis *hy5* mutants (Oyama et al., 1997; Cluis et al., 2004), whereas it may be repressed in *hy5-215 slr-1* mutants due to the gain-of-function mutation of *SLR/IAA14*. Therefore, the similar tolerance phenotypes between *hy5-215 slr-1* and *hy5-215* also suggest that auxin signaling may not be responsible for the Pi-deficiency tolerance in *hy5-215*.

### External Pi Acquisition Is Not Involved in Pi-Deficiency Tolerance of *hy5-215*

**Figure 6:**
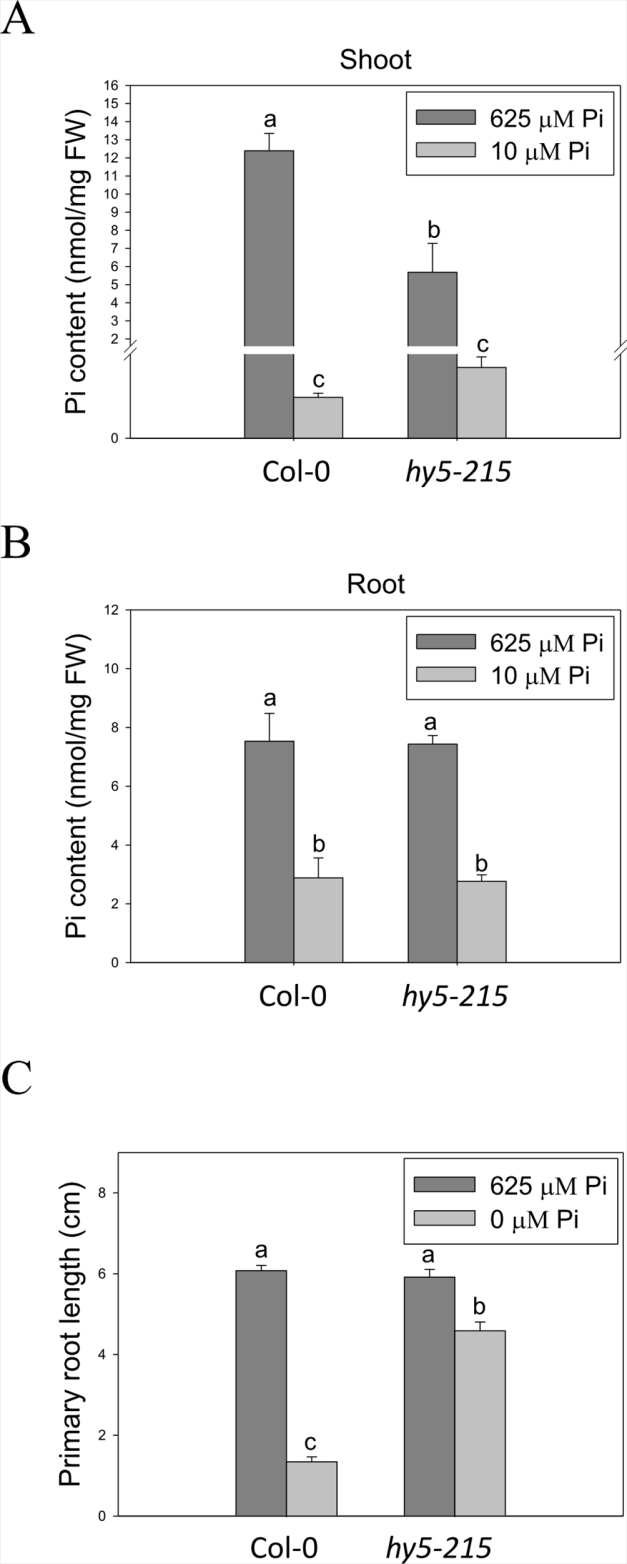
Pi content in wild-type and mutant seedlings in response to Pi treatment. **(A)** Soot Pi content in Col-0 and *hy5-215*. **(B)** Root Pi content in Col-0 and *hy5-215*. **(C)** PR length in Col-0 and *hy5-215* when Pi was sufficient or absent. The seedlings were grown on 1/2 MS medium with 625, 10, or 0 μM Pi for 10 days. Data represent means ± SE of four independent experiments. Different letters above the bars indicate statistically significant differences among the means based on Two-way ANOVA followed by Fisher's LSD tests (*P < *0.05).

Enhancement of Pi inﬂux through induction of high-afﬁnity Pi transporter genes is one of the conserved strategies evolved by plants to optimize their growth in response to Pi limitation. There are nine genes encoding *PHT* homologs (*PHT1;1–PHT1;9*) in the Arabidopsis genome. Most of the *PHT1* family genes are strongly induced by low Pi treatment within the ﬁrst 12 hours (Bayle et al., 2011; Nussaume et al., 2011). Functional studies show a major role for PHT1 in Pi acquisition in roots from Pi-deficient environment; however, some of the PHTs are also required for Pi mobilization (PHT1;5, PHT1;8 and PHT1;9), ﬂower development (PHT1;6), and Pi uptake in Pi replete condition (PHT1;1 and PHT1;4) (Nussaume et al., 2011; Nagarajan et al., 2011; Lapis-Gaza et al., 2014). In this study, we found that expression of *PHT1* family genes was lower in *hy5-215* shoots than in the WT, suggesting *hy5-215* may not be as deficient as WT under low Pi treatment (**Supplementary Figure 7**). However, three *PHT1* genes, *PHT1;5*, *PHT1;8,* and *PHT1;9*, were induced in a higher level in *hy5-215* roots under both sufficient and deficient conditions (**Supplementary Figure 8** and **Table 1**). To demonstrate whether the higher *PHT1* gene expression in *hy5-215* roots can increase Pi uptake and subsequently contribute to Pi-deficiency tolerance, the free Pi content was measured. Unexpectedly, a great reduction of Pi level was found in *hy5-215* shoots under Pi sufficient condition (**Figure 6A**). Although Pi content was slightly higher in *hy5-215* shoots than in WT under Pi deficiency, no statistically significant difference (*P <*0.05) was found between them. In addition, there was no significant difference between WT and *hy5-215* in roots (**Figure 6B**). The results indicated that the elevated amounts of *PHT1* transcripts in *hy5-215* roots might not or only partially contribute to Pi deficiency tolerance of *hy5-215*. To verify this finding, we cultured WT and *hy5-215* plants on Pi-free media. The *hy5-215* plants exhibited similar growth on Pi-free medium and on Pi-deficient medium containing 10 µM Pi. The PR length of *hy5-215* grown on Pi-free medium was only slightly diminished compared to that of plants grown on Pi-deficient medium (**Figure 6C** and **Supplementary Figure 9A**). A similar phenotype was found in *35S:BBX32-SRDX* (**Supplementary Figure 9B**). Altogether, these results indicate that the induction of *PHT1;5*, *PHT1;8*, and *PHT1;9* in *hy5-215* roots may be involved in Pi translocation from roots to shoots but not Pi uptake from roots, and the root tolerant phenotype of *hy5-215* to Pi deficiency is not related to extracellular Pi acquisition. Furthermore, it also suggests that the pre-accumulated Pi in seeds during seed development is sufficient to support *hy5-215* growth at the early stages of Pi deficiency.

### Lower Level of Pi Deficiency-Inducible Membrane Glycolipids in *hy5-215*

Since Pi deficiency tolerance of *hy5-215* was not due to Pi acquisition, we investigated Pi use efficiency in the mutant and wild type. Improvement of Pi utilization efficiency helps plants to conserve internal Pi and can involve the recycling of Pi from senescent tissues and the replacement of Pi from cellular structures or metabolic processes by alternative non-Pi compounds (Kobayashi et al., 2006; Rose et al., 2013). Membrane lipid remodeling, in which phospholipids are hydrolyzed and replaced by non-phosphorus glycolipids, such as sulfoquinovosyldiacylglycerol (SQDG) and digalactosyldiacylglycerol (DGDG), is a representative mechanism of Pi recycling, which improves Pi use efficiency (Kobayashi et al., 2006; Nakamura, 2013). Therefore, we analyzed the expression of genes involved in hydrolysis of phospholipids, non-specific phospholipase C4 gene (*NPC4*), and synthesis of SQDG and DGDG including *SQD1*, *SQD2*, *MGDG2*, and *MGDG3* (monogalactosyldiacylglycerol synthetic genes) in the WT and *hy5-215*. All the analyzed genes were induced by Pi deficiency, but the expression levels were lower in *hy5-215* than in the WT (**Supplementary Figures 10A–E**). The lipid composition calculated as the ratio of DGDG and PC (phosphatidylcholine), one of the major membrane phospholipids, is used as a marker to indicate a Pi-deficient state (Kobayashi et al., 2006). Enhancement of the DGDG/PC ratio represents an increase in DGDG biosynthesis to replace membrane phospholipids in response to Pi deficiency. A lower ratio of DGDG/PC was found in *hy5-215* under Pi-deficient conditions (**Supplementary Figure 10F**), indicating that the increased tolerance to Pi deficiency in *hy5-215* mutants is not caused by increased free Pi from phospholipids.

### Light Quality Is Involved in Regulation of Pi Deficiency Response

**Figure 7:**
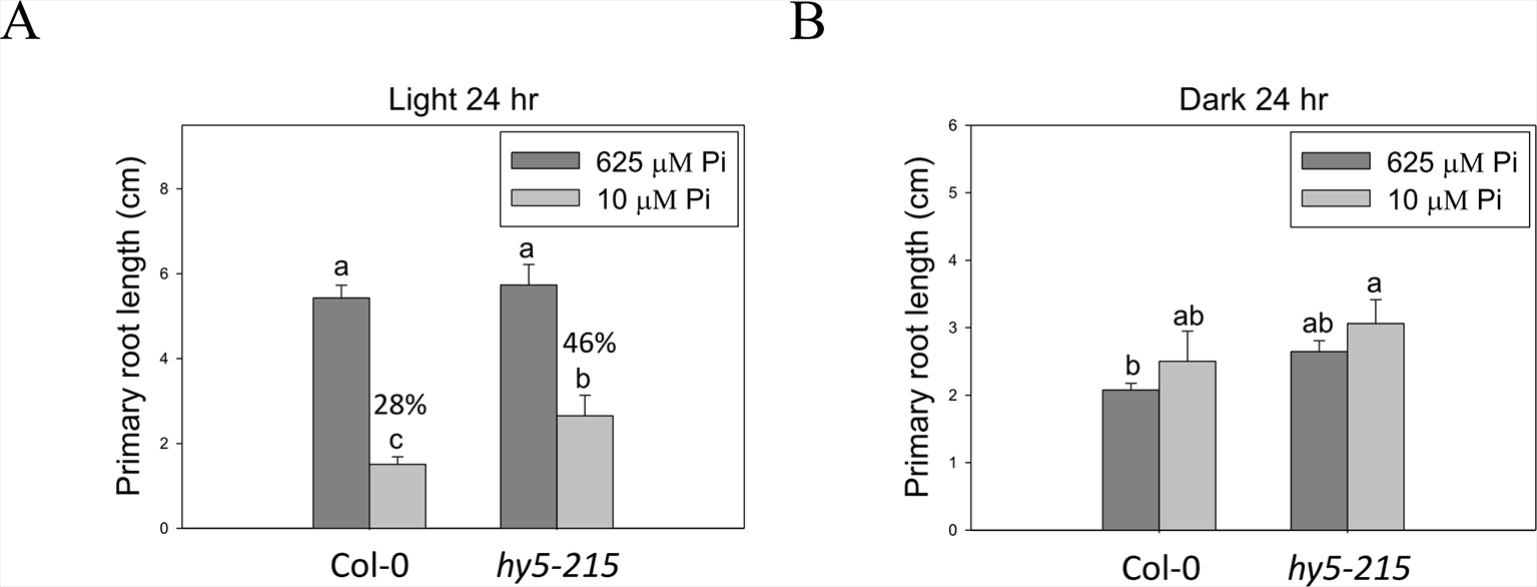
Effect of light on Pi-deficiency tolerance in Arabidopsis. The seedlings were grown on 1/2 MS media with 625 or 10 μM Pi under continuous light **(A)** or dark **(B)** treatments. The PR length was measured after 10 days of growth. Data represent means ± SE of four independent experiments. Different letters above the bars indicate statistically significant differences among the means based on Two-way ANOVA followed by Fisher's LSD test (*P < *0.05).

Because HY5 acts as an integrator of different light signaling pathways downstream of multiple photoreceptor families and regulates photomorphogenesis (Cluis et al., 2004), we examined the effect of light on *hy5-215* tolerance to Pi deficiency. When the seedlings were grown in Pi-deficient conditions under continuous white light, WT and *hy5-215* PR lengths were 28% and 46% of PR lengths under Pi-sufficient conditions, respectively (**Figure 7A**). Under continuous dark, there were no significant differences in PR growth between WT and *hy5-215* (**Figure 7B**). These results, together with the results from long-day treatments (16 h light/8 h dark; **Figure 4B**), indicate that increased light irradiation time inhibits Arabidopsis PR growth in Pi-deficient conditions. Therefore, light may play a role in *hy5-215* tolerance to Pi deficiency.

**Figure 8:**
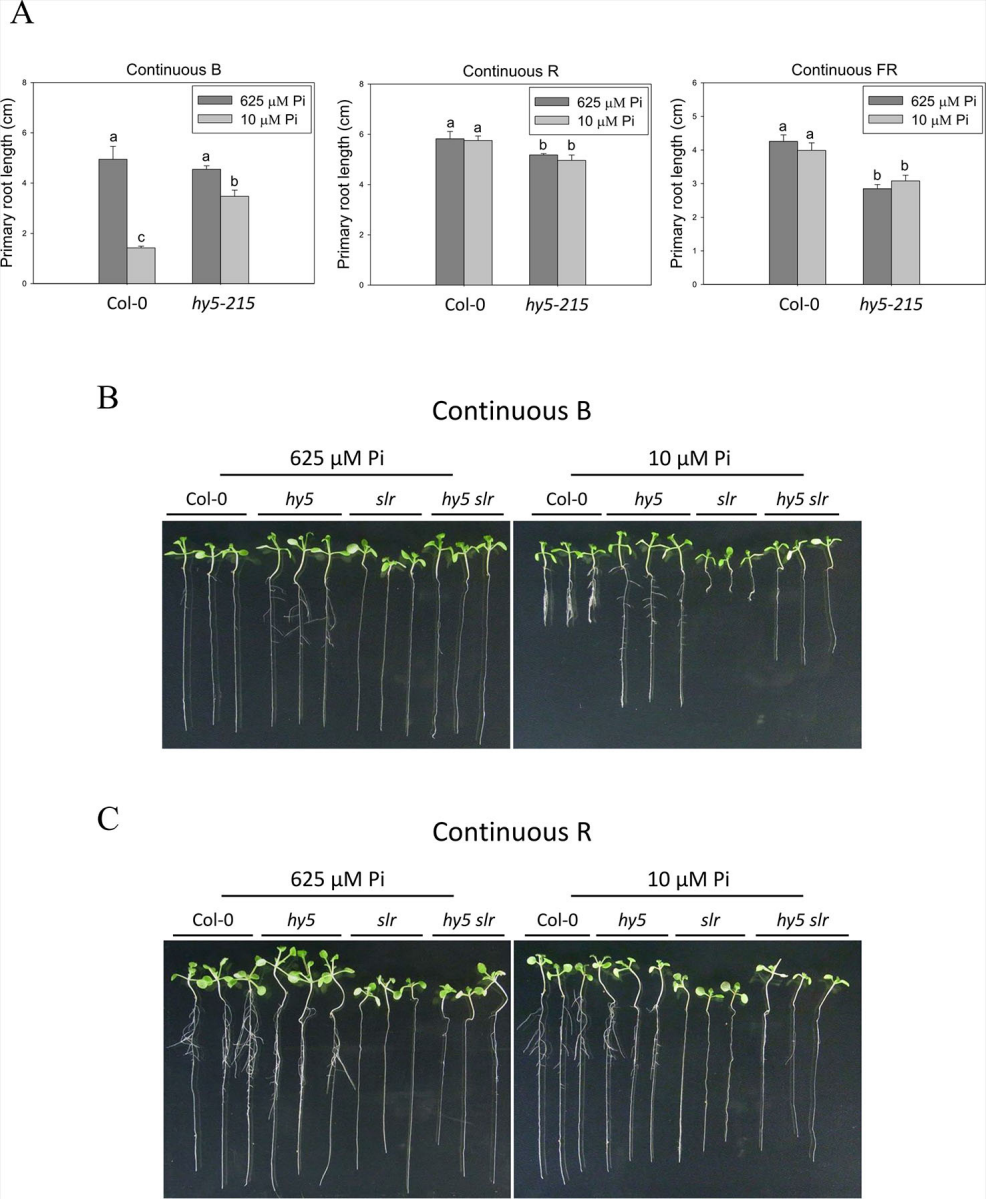
Effect of light quality on primary root length in Arabidopsis. The Col-0 and *hy5-215* seedlings were grown on 1/2 MS media with 625 or 10 μM Pi under continuous blue (B), red (R), or far red (FR) light treatments, respectively. The PR length was measured after 10 days of growth **(A)**. The Col-0, *hy5-215*, *slr*, and *hy5-215slr-1* seedlings grown on Pi-sufficient and Pi-deficient media under continuous B **(B)** and R **(C)** light treatments. Data represent means ± SE of four independent experiments. Different letters above the bars indicate statistically significant differences among the means based on Two-way ANOVA followed by Fisher's LSD test (*P < *0.05).

**Figure 9:**
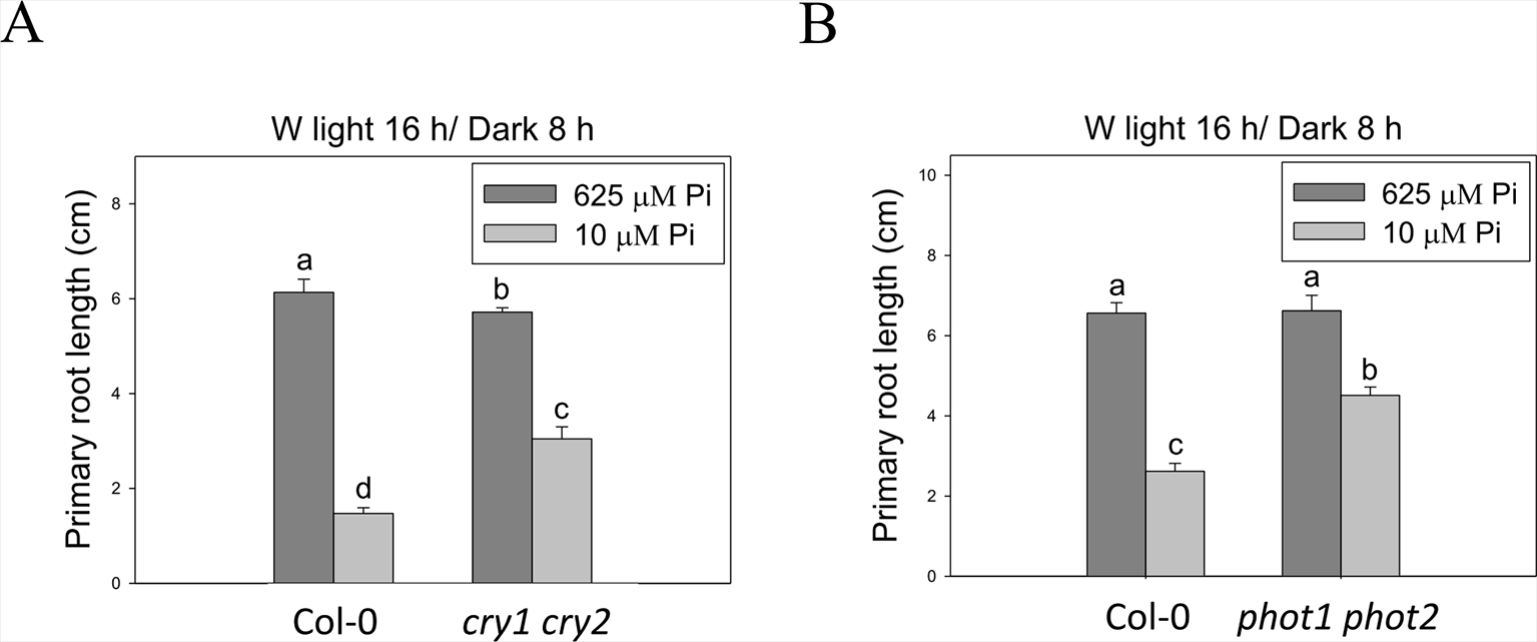
Primary root length of wild-type and blue light receptor mutant seedlings in response to Pi treatment. The blue light receptor mutants, *cry1 cry2* **(A)** and *phot1 phot2* **(B)**, were grown on Pi-sufficient and Pi-deficient media under long-day condition (16 h light/8 h dark). The PR length was measured after 10 days of growth. Data represent means ± SE of four independent experiments. Different letters above the bars indicate statistically significant differences among the means based on Two-way ANOVA followed by Fisher's LSD test (*P < *0.05).

To better understand light effects on Pi-deficiency tolerance, Arabidopsis plants were grown under continuous blue (B), red (R), and far-red (FR) light. PR growth was inhibited by Pi deficiency in the WT under continuous B light (**Figures 8A, B**). In contrast, the same level of inhibition by Pi deficiency under B light was not observed in *hy5-215*. Interestingly, PR growth was not inhibited by Pi deficiency in the WT and all tested mutants when grown under continuous R and FR irradiation (**Figures 8A, C**). These results indicate that the root tolerant phenotype of *hy5-215* to Pi deficiency is negatively regulated by B light. To further confirm this finding, the B light receptor mutants, *cry1 cry2* and *phot1 phot2*, were examined under Pi deficiency (Fankhauser and Chory, 1999; Kinoshita et al., 2001). Indeed, a tolerant phenotype to Pi deficiency was found in these two mutants (**Figure 9**). Therefore, the tolerance of *hy5-215* to Pi deficiency likely results from blockage of B light responses, and the tolerance mechanism may be related to enhancement of internal Pi recycling or utilization efficiency but not external Pi acquisition due to the observation of *hy5-215* tolerance on Pi-free condition. Our findings may provide valuable insights for developing Pi deficiency-tolerant crops in the future. Furthermore, light quality-regulated responses to Pi deficiency may allow indoor plant growers to reduce Pi fertilizer application through proper illumination.

### Identification of Possible Candidate Genes Responsible for Pi-Deficiency Tolerance in *35S:BBX32-SRDX* and *hy5-215*

To determine the Pi-deficiency tolerance mechanism of *hy5-215* and *35S:BBX32-SRDX*, we performed several microarray experiments using mRNAs from shoots and roots. The transcriptomic analysis indicates a large number of genes show similar expression pattern between *hy5-215* and *35S:BBX32-SRDX* samples (**Supplementary Figure 11** and **Tables 1**–**3**). Consistent with previous reports, the well-known PSI genes were up-regulated in the WT under Pi deficiency. However, the expression levels of most PSI genes were significantly lower in *hy5-215* and *35S:BBX32-SRDX*, including genes encoding high-affinity Pi transporters, ribonucleases, acid phosphatases, lipid remodeling, and anthocyanin synthesis enzymes (**Supplementary Table 1**). Previously reported Pi deficiency-responsive TF genes in Arabidopsis mainly belong to the MYB and WRKY families (Rubio et al., 2001; Bustos et al., 2010; Yeh and Ohme-Takagi, 2015). In this study, various TF genes, including *MYB*, *WRKY*, *AP2/ERF*, *bHLH*, *C2H2ZnF*, and *MADS-box*, were up-regulated or down-regulated in *hy5-215* and *35S:BBX32-SRDX* under Pi-deficient conditions (**Supplementary Table 2**), suggesting their possible roles in the Pi deficiency tolerance. Liu et al. (2017) have reported that HY5 negatively regulates expression of *PHR1* and its downstream PSI genes, and *hy5* mutation increases Pi and anthocyanin contents. According to their data, the longer root phenotype of *hy5* under phosphate starvation may result from the increased PSRs and Pi content. Although their root phenotypes of *hy5* are similar to our results, PSI gene expression and PSRs are different between two studies. The expression of PSI genes and anthocyanin content were lower in the *hy5-215* mutant in our study (**Supplementary Figures 3–5** and **Table 1**), which is consistent with previous reports that the expression of anthocyanin biosynthesis genes and anthocyanin accumulation are reduced in *hy5* (Lee et al., 2007; Jeong et al., 2010; Shin et al., 2013). In addition, they reported an increase in total Pi content in *hy5-215* seedlings under Pi sufficient condition; however, we found that free Pi content is significantly lower in *hy5-215* shoots (**Figure 6**). Our results clearly show that the *hy5-215* tolerant phenotype to Pi starvation is unlikely to be related to external Pi uptake because of similar growths of *hy5-215* on Pi-deficient and Pi-free conditions (**Figure 6C**). Further study is required to address whether these inconsistencies result from different growth conditions or plant tissues selected for the experiments. For example, we performed all the experiments by using both shoot and root samples, but Liu et al. (2017) used whole seedlings. Many genes or metabolisms show distinct expression patterns in specific tissues or organs; therefore, analysis using a mixture of shoot and root samples may not detect their real state in each organs. Sakuraba et al. (2018) reported that HY5 can activate high-affinity Pi transporter genes including *PHT1;1*, *PHT1;4*, *PHT1;5*, *PHT1;8*, and *PHT1;9*. Overexpression of *HY5* promotes Pi uptake activity, while loss-of-function of *HY5* reduces the activity. These results to some degree support our findings but not Liu's (Liu et al., 2017). However, Sakuraba et al. (2018) also used Arabidopsis whole seedlings as materials. Their results may mainly reflect the status of the *PHT1* gene expression and Pi content in shoots because the biomass of shoot is much higher than that of root (around 3–5 times in our study). As shown in **Supplemental Figure 7**, the expression patterns of *PHT1;1*, *PHT1;4*, *PHT1;5*, *PHT1;8*, and *PHT1;9* in *hy5-215* shoots are similar to the results shown by Sakuraba et al. (2018). However, the expression of those genes in *hy5-215* roots are opposite. It suggests that the regulation of the *PHT1* genes may be different in shoots and roots. In addition, they concluded that the HY5-mediated activation of Pi uptake is red-light-dependent. However, in this study, the HY5-regulated PR growth inhibition is dependent on blue light treatment. The results indicate that light quality may play different roles in regulating different PSRs.

Unexpectedly, a significant number of photosynthesis-related and chlorophyll synthesis genes were down-regulated in roots but not shoots of *hy5-215* and *35S:BBX32-SRDX* (**Supplementary Figure 11** and **Table 3**). Reduction of photosynthesis is a well-known PSR due to the reduction of Pi consumption for ATP synthesis (Wu et al., 2003; Kang et al., 2014). Plant roots can accumulate chlorophyll and turn green under light illumination. The green roots are supposed to have photosynthetic ability as green leaves (Kobayashi et al., 2012). We therefore considered whether the Pi-deficiency tolerance of *hy5-215* is related to down-regulation of photosynthesis-related and chlorophyll synthesis genes, which may induce lower Pi consumption by decreasing photosynthesis in *hy5-215* roots. *GLK1* and *GLK2* have been shown to regulate expression of various photosynthetic genes in Arabidopsis roots (Kobayashi et al., 2012; Kobayashi et al., 2013). In addition, it was reported that the roots of *35S:GLK1* accumulate much chlorophyll and are hypersensitive to Pi deficiency (Kang et al., 2014).We thus examined whether the *glk* mutants also show tolerance to Pi deficiency (Nagatoshi et al., 2016). The similar PR lengths between WT and *glk* mutants indicate *GLK1* and *GLK2* may not be involved in Pi-deficiency tolerance (**Supplementary Figures 12A, C**). We further investigated the overexpression lines of *GLK1* and *GLK2* in *hy5-215* background (*35S:GLK1 hy5-215* and *35S:GLK2 hy5-215*), which have a recovered chlorophyll content as WT (Kobayashi et al., 2012). The *35S:GLK1 hy5-215* and *35S:GLK2 hy5-215* plants exhibited longer PR lengths under Pi deficiency similar to *hy5-215* (**Supplementary Figure 12B**), suggesting that tolerance of *hy5-215* to Pi deficiency may not be related to chlorophyll content and photosynthetic activity.

The photosynthetic ability of *hy5-215* and WT plants was also compared in shoots, although photosynthetic gene expression in shoots was not significantly different between *hy5-215* and WT under both sufficient and deficient conditions. As shown in **Supplementary Figure 13**, the maximum quantum yield of photosystem II (Fv/Fm) and the actual quantum yield of photosystem II under light (YII) were reduced in the cotyledons of both WT and *hy5-215* in response to Pi deficiency. Although the measurement of Fv/Fm and Y_II_ of *hy5-215* under Pi sufficient treatment were lower than those of WT, there was no significant difference between WT and *hy5-215* in response to Pi depletion. In addition, Fv/Fm and Y_II_ in the true leaves of WT and *hy5-215* were not affected by our low Pi treatment. These data indicate that the tolerance of *hy5-215* to Pi deficiency is not related to photosynthetic ability (**Supplementary Figure 13**). Enhancement of RNA degradation by ribonucleases or phosphatases is another well-known mechanism against Pi deficiency. Therefore, the significant down-regulation of a large number of photosynthesis and chlorophyll biosynthesis genes in *hy5-215* roots might, to some degree, contribute to Pi deficiency tolerance through reduction of Pi consumption for biosynthesis of RNA rather than for photosynthesis.

### Identification of Genes Responsible for Blue Light-Regulated Primary Root Growth Inhibition in Response to Pi Deficiency

**Figure 10:**
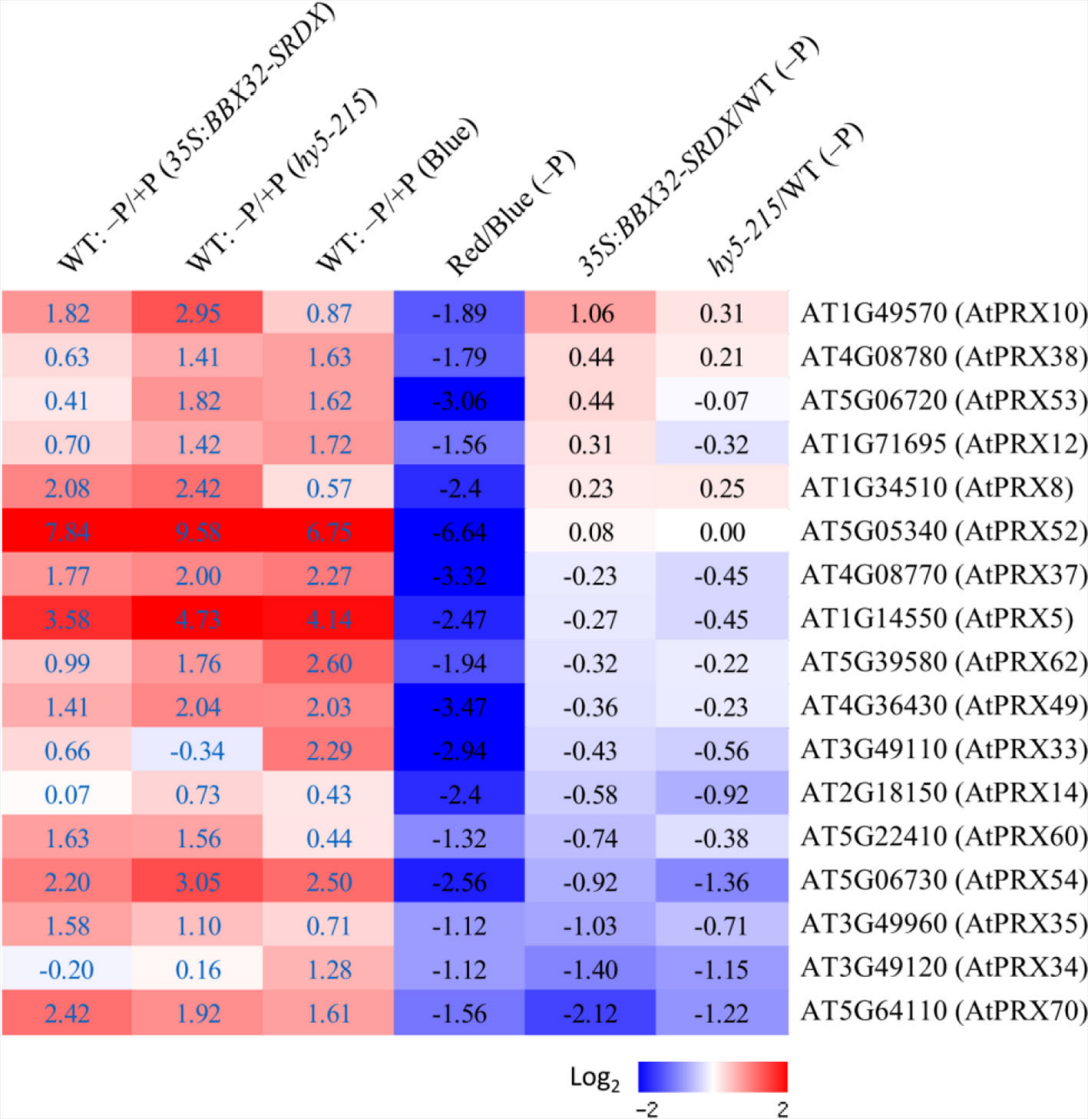
Expression of Pi deficiency-inducible Class III PRX genes under different light treatments. Expression levels of Class III PRX genes were analyzed under different conditions or in different transformants/mutants. The log2 fold change was calculated. Columns 1–3 represent expression levels of PRX genes induced by Pi deficiency in WT roots from different sets of transcriptome experiments (*35S:BBX32-SRDX*, *hy5-215*, and B light, respectively). Columns 4 to 6 represent the expression ratio of PRX genes under criteria of R/B, *35S:BBX32-SRDX*/WT, and *hy5-215*/WT in response to Pi deficiency.

Because the PR growth inhibition in response to Pi deficiency only appears under B light illumination, the comparison of root transcriptomes between Arabidopsis WT seedlings grown under B and R light treatments was performed to identify candidate genes responsible for Pi deficiency tolerance. Genes encoding enzymes involved in different metabolic pathways were analyzed. Metabolic pathway analysis indicates that numerous class III peroxidase (PRX) genes were significantly down-regulated in response to Pi deficiency under R light condition (**Supplementary Figure 14**). It has been reported that PR growth inhibition in response to Pi deficiency is mediated by PRX activity at Arabidopsis root tips (Balzergue et al., 2017). Class III PRXs are involved in cell wall tightening by catalyzing crosslinking between some polysaccharides or proteins, subsequently leading to inhibition of cell expansion (Balzergue et al., 2017). Therefore, we next examined the expression levels of the 73 Class III PRX genes in the Arabidopsis genome and identified that 17 of them are Pi deficiency-inducible in three different sets of microarray experiments, including the root transcriptomes of WT corresponding to those of *35S*:BBX32-SRDX and *hy5-215*, and the root transcriptome of WT under B light treatment (columns 1–3 in **Figure 10**). Interestingly, all of these PRX genes were down-regulated or not induced by Pi deficiency under R light illumination. The values of log_2_ fold changes from R to B light (R/B) indicate the PRX genes were under-expressed (column 4 in **Figure 10**). In addition, most of these genes show a similar expression pattern in *35S*:BBX32-SRDX and *hy5-215* when compared to the WT (columns 5 and 6 in **Figure 10**). These findings imply that the long PR lengths of *35S*:BBX32-SRDX and *hy5-215* under Pi deficiency may, at least partially, result from lower cell wall stiffness caused by the reduction of PRX gene expression from the blockage of B-light responses. Therefore, the phenotype of *35S*:BBX32-SRDX and *hy5-215* may represent Pi-deficiency insensitivity but not tolerance. However, in addition to longer PR lengths, the shoot sizes and seedling FWs of *35S*:BBX32-SRDX and *hy5-215* are bigger and higher, respectively, than those of WT under Pi-deficient condition. The results suggest potential Pi-deficiency tolerance in *35S*:BBX32-SRDX and *hy5-215*.

In the pathway proposed by Balzergue et al. (2017), Pi deficiency post-transcriptionally activates STOP1 and subsequently induces *ALMT1* expression leading to malate efﬂux to the apoplast. In turn, Fe chelation by malate and Fe redox cycling mediated by LPR1 ferroxidase activity inhibit cell elongation in PR tip through stimulation of peroxidase-dependent cell wall stiffening. Although the PR growths and suppression of PRXs in *35S*:BBX32-SRDX and *hy5-215* are similar to *stop1* and *almt1*, the expression levels of *STOP1* and *ALMT1* were not reduced in *35S*:BBX32-SRDX, *hy5-215*, or R/B condition. These results indicate that BBX32 and HY5 do not act upstream of STOP1 and ALMT1 and B light regulates pathways distinct to the STOP1–ALMT1-dependent pathway. Altogether, this study indicates that BBX32 and HY5 are involved in the regulation of PSRs and that B light may regulate PR growth inhibition in response to Pi deficiency by the activation of Class III PRX genes. Moreover, a novel mechanism other than the known PSRs may account for the tolerance of *35S*:BBX32-SRDX and *hy5-215* to Pi deficiency. Further studies, such as metabolome analysis, are required to comprehensively address the roles of BBX32, HY5 and light quality in Pi deficiency responses or tolerance.

## Materials and Methods

### Plant Materials and Growth Conditions

Twenty-thousand T2 seeds of Arabidopsis CRES-T lines were used to screen candidates with altered phenotypes in response to Pi deficiency. The seeds were sterilized by 0.5% sodium hypochlorite for 10 minutes and then washed with sterile distilled water for at least three times before sowing on 1/2 Murashige and Skoog (MS) media with 10 μM Pi (K_2_HPO_4_). The CRES-T seedlings were grown on vertical plates for 10 days. Several phenotypes affected by Pi deficiency including plant size, primary root length, and anthocyanin accumulation were observed or measured.

The surface-sterilized seeds of *Arabidopsis thaliana* wild type [ecotypes Columbia (Col-0)], mutants (*hy5-215*, *slr-1*, *hy5-215 slr-1*, *glk1*, *glk2*, *glk1 glk2*, *cry1 cry2*, *phot1 phot2*), and transformants (*35S:BBX32-SRDX*, *35S:BBX32*, *35S:GLK1 hy5-215*, and *35S:GLK2 hy5-215*) were sown on 1/2 MS agar plates containing 625 μM KH_2_PO_4_ (Pi sufficient) or 10 μM KH_2_PO_4_ (Pi deficient). Each experiment used 10 plants and was replicated three to four times. The seedlings were grown at 22°C and illuminated with 100–125 μmol m^-2^ s^-1^ white light for 16 hours per day or with blue (B), red (R), and far-red (FR) light for 24 hours. For determination of primary root (PR) length and fresh weight, the seedlings were cultured on vertical and horizontal plates for 10 and 14 days, respectively. The seedlings were then collected for photographs, measurement of PR length and fresh weight, and further experiments.

### Quantiﬁcation of Anthocyanin Content

The shoots of 10-day-old seedlings were frozen in liquid nitrogen, ground into a powder, and then re-suspended in an extraction buffer containing 45% methanol and 5% acetic acid. The supernatant was taken after centrifugation at 12,000 rpm for 10 minutes. Anthocyanin content was calculated by the absorbance at 530 and 637 nm as described previously (Matsui et al., 2004).

### Determination of Acid Phosphatase Activity

The histochemical staining of acid phosphatase activity was performed according to the method described by Yu et al. (2012) with some modifications. The roots of 10-day-old seedlings were overlaid with a 0.1% agar solution containing 0.01% 5-bromo-4-chloro-3-indolyl phosphate (BCIP). The acid phosphatase activity indicated by blue color on the root surface was observed and photographed after 6 to 24 hours.

### Determination of Lipid Composition

Seedlings were grown on 1/2 MS medium with 625 μM Pi for 10 days and then transferred to 1/2 MS medium with 625 μM Pi or 10 μM Pi for 10 days. Samples were collected and immediately frozen in liquid nitrogen. Lipids were then extracted and analyzed by the method described by Kobayashi et al. (2006).

### RNA Isolation, Reverse-Transcription Quantitative PCR (RT-qPCR), and Microarray Analyses

Total RNA was extracted by using the RNeasy Plant Mini kit (QIAGEN, Hilden, Germany) following the manufacturer's instructions. One microgram (1 μg) of total RNA was subjected to ﬁrst-strand cDNA synthesis using the PrimeScript RT reagent kit (Takara). Quantitative RT-qPCR was performed by the SYBR green method using the ABI7300 real-time PCR system (Applied Biosystems) as described previously (Mitsuda et al., 2005). The *UBQ1* gene was used as an internal control. The microarray experiments and the data analysis were conducted by the method described by Mitsuda et al. (2005). Three biological replicates were performed for the microarray studies except for the experiments conducted under blue and red light treatments (two biological replicates), which aim to isolate candidate genes with similar expression pattern to the microarray experiments performed in *35S:BBX32-SRDX* and *hy5-215*. The expression of the Class III PRX candidate genes isolated from the transcriptomes in response to blue and red light treatments was confirmed by the third biological replicates using RT-qPCR. For confirming the other transcriptomes, the RT-qPCR data are averages from three or four biological replicates.

### Measurement of Photosynthetic Activity

The maximum quantum yield of photosystem II (F_v_/F_m_) and actual quantum yield of photosystem II in light (Y_II_) of cotyledons and true leaves were measured according to the method described by Kobayashi et al. (2013).

### Statistical Analysis

All the experiments were performed in a completely randomized design. Data on root length (cm) and seedling fresh weight (mg) were recorded after growth for 10 and 14 days, respectively. Two-way analysis of variance (ANOVA) and mean comparisons using least significant difference (LSD) tests were conducted. Data represent means of three or four independent experiments. Different letters above bars indicate statistically significant differences (*P < *0.05).

## Data Availability Statement

Arabidopsis Genome Initiative numbers described in this article are as follows: ACP5 (At3g17790), CHS (At5g13930), DFR (At5g42800), GLK1 (At2g20570), GLK2 (At5g44190), HY5 (At5g11260), IPS1 (At3g09922), LDOX (At4g22880), MGD2 (At5g20410), MGD3 (At2g11810), MYB75 (At1g56650), MYB90 (At1g66390), NPC4 (At3g03530), PHT1;2 (At5g43370), PHT1;3 (At5g43360), PHT1;4 (At2g38940), PHT1;5 (At2g32830), PHT1;7 (At3g54700), PHT1;8 (At1g20860), PHT1;9 (At1g76430), RNS1 (At2g02990), SLR/IAA14 (At4g14550), SQD1 (At4g33030), SQD2 (At5g01220) and UF3GT (AT5G54060). The microarray data referred in this study is available from NCBI GEO under the accession number GSE139100.

## Author Contributions

C-MY and MO-T designed the experiments and coordinated the project. C-MY performed most of the experiments. KK and SF conducted the analysis of lipid composition and photosynthetic activity. NM performed the microarray experiments. HF created the *slr* and *hy5 slr* mutants, propagated the seeds, and provided valuable comments on the manuscript. C-MY analyzed the data. C-MY wrote the manuscript and MO-T revised the manuscript.

## Funding

This work was supported by JSPS KAKENHI Grant Numbers 12F02210, 25291055.

## Conflict of Interest

The authors declare that the research was conducted in the absence of any commercial or financial relationships that could be construed as a potential conflict of interest.

## Abbreviations

B, blue; BBX32, B-BOX32; DGDG, digalactosyldiacylglycerol; FR, far-red; hps, hypersensitive to phosphate starvation; HY5, ELONGATED HYPOCOTYL 5; LR, lateral root; MGDG, monogalactosyldiacylglycerol; NPC4, non-specific phospholipase C4; PC, phosphatidylcholine; PHL1, PHR1-like 1; PHO1, PHOSPHATE1; PHR1, PHOSPHATE STARVATION RESPONSE 1; Pi, inorganic phosphate; PR, primary root; PSI, phosphate starvation-induced; PSR, phosphate starvation response; R, red; slr-1, solitary-root-1; SQDG, sulfoquinovosyldiacylglycerol; TF, transcription factor; WT, wild type.
