# Supplementary material for: Blue Light Regulates Phosphate Deficiency-Dependent Primary Root Growth Inhibition in Arabidopsis
Source: Front Plant Sci. 2020 Jan 31;10:1803. doi: 10.3389/fpls.2019.01803 (PMC7005603; doi:10.3389/fpls.2019.01803)
Supplement: Supplementary file 1 [file Presentation_1.pdf]

Supplementary Material

Blue light regulates phosphate deficiency-dependent primary root growth inhibition in Arabidopsis

Chuan-Ming Yeh[1,2,3], Koichi Kobayashi[4], Sho Fujii[4], Hidehiro Fukaki[5], Nobutaka Mitsuda[2], Masaru Ohme-Takagi[1,2*]

[1] Graduate School of Science and Engineering, Saitama University, Saitama, Japan
[2] Bioproduction Research Institute, National Institute of Advanced Industrial Science and Technology (AIST), Tsukuba, Ibaraki, Japan
[3] Institute of Tropical Plant Sciences and Microbiology, College of Bioscience and Biotechnology, National Cheng Kung University, Tainan, Taiwan
[4] Graduate School of Arts and Sciences, The University of Tokyo, Tokyo, Japan
[5] Graduate School of Science, Kobe University, Kobe, Japan

CORRESPONDENCE:
Professor Masaru Ohme-Takagi
mtakagi@mail.saitama-u.ac.jp

Supplementary Note

Anthocyanin accumulation is one of the typical symptoms induced by Pi starvation in plants (Trull et al. 1997, Ticconi et al. 2001). To confirm tolerance to Pi-deficient conditions in *hy5-215*, anthocyanin accumulation in response to Pi deficiency was examined. The leaves of the 14-day-old seedlings grown on Pi-deficient and Pi-sufficient media were collected for anthocyanin measurements. Anthocyanin content significantly increased in response to Pi deficiency in both wild type (WT) and *hy5-215*; however, the content in *hy5-215* was only one-third of that in WT (Supplementary Figure 3A, B). In addition, the expression of genes involved in anthocyanin biosynthesis was analyzed. The Arabidopsis MYB TF family genes, *MYB75* and *MYB90*, have been reported to be involved in the regulation of anthocyanin biosynthesis (Borevitz et al. 2000). The transcripts of *MYB75* and *MYB90* were significantly induced by Pi deficiency, but the level was much lower in *hy5-215* than in WT (Supplementary Figure 3C, D). Similar results were obtained in the expression of the anthocyanin biosynthesis genes coding for chalcone synthase (CHS), dihydroflavonol 4-reductase (DFR), leucoanthocyanidin dioxygenase (LDOX), and UDP glucose flavonoid 3-glucosyltransferase (UF3GT) (Solfanelli et al. 2006, Sakuraba et al. 2010). Although some of the anthocyanin biosynthesis genes were previously shown to be down-regulated in *hy5* under normal conditions (Lee et al. 2007, Jeong et al. 2010, Shin et al. 2013), a much bigger difference of the expression level between low-Pi treated *hy5-215* and WT was found (Supplementary Figure 4). Taken together, the data somehow suggest *hy5-215* is tolerant to Pi deficiency.

Activation or secretion of acid phosphatases, ribonucleases and organic acids are responsible for scavenging extracellular Pi from insoluble organic complexes. In addition, the activities of acid phosphatases and ribonucleases also contribute to release Pi from intracellular organic Pi sources (Raghothama 2000, Poirier and Bucher 2002). Then, we analyzed the expression of *RNS1* and *ACP5*, which encode a ribonuclease and purple acid phosphatase (PAP), respectively. The expression of *RNS1* and *ACP5* was significantly induced by Pi deficiency in the WT, but their transcript levels were much lower in *hy5-215* than in the WT (Supplementary Figure 5A-D). In addition, acid phosphatase activity, monitored by staining with 5-bromo-4-chloro-3-indolyl phosphate (BCIP), was significantly reduced in *hy5-215* compared to the WT under Pi-deficient conditions (Supplementary Figure 5E). These results indicate that *hy5-215* suffers less stress in Pi-deficient conditions and release less free Pi from organic molecules by ribonucleases or PAP, compared with the WT.

Supplementary references

Trull M.C., Guiltinan M.J., Lynch J.P., Deikman J. (1997) The responses of wild-type and ABA mutant Arabidopsis plants to phosphorus starvation. *Plant Cell Environ.* 20, 85-92.

Ticconi C.A., Delatorre C.A., Abel S. (2001) Attenuation of phosphate starvation responses by phosphite in Arabidopsis. *Plant Physiol.* 127: 963-972.

Borevitz J.O., Xia Y., Blount J., Dixon R.A., Lamb C. (2000) Activation tagging identifies a conserved MYB regulator of phenylpropanoid biosynthesis. *Plant Cell* 12: 2383-2394.

Solfanelli C., Poggi A., Loreti E., Alpi A., Perata P. (2006) Sucrose-specific induction of the anthocyanin biosynthetic pathway in Arabidopsis. *Plant Physiol.* 140: 637-646.

Sakuraba Y., Yokono M., Akimoto S., Tanaka R., Tanaka A. (2010) Deregulated chlorophyll b synthesis reduces the energy transfer rate between photosynthetic pigments and induces photodamage in *Arabidopsis thaliana*. *Plant Cell Physiol.* 51: 1055-1065.

Lee J., He K., Stolc V., Lee H., Figueroa P., Gao Y. et al. (2007) Analysis of transcription factor HY5 genomic binding sites revealed its hierarchical role in light regulation of development. *Plant Cell* 19: 731-749.

Jeong S.W., Das P.K., Jeoung S.C., Song J.Y., Lee H.K., Kim Y.K. et al. (2010) Ethylene suppression of sugar-induced anthocyanin pigmentation in Arabidopsis. *Plant Physiol.* 154: 1514-1531.

Shin D.H., Choi M., Kim K., Bang G., Cho M., Choi S.B. et al. (2013) HY5 regulates anthocyanin biosynthesis by inducing the transcriptional activation of the MYB75/PAP1 transcription factor in Arabidopsis. *FEBS Lett.* 587: 1543-1547.

Poirier Y., Bucher M. (2002) Phosphate transport and homeostasis in Arabidopsis. *Arabidopsis Book* 1: e0024.

Raghothama K.G. (2000) Phosphate transport and signaling. *Curr. Opin. Plant. Biol.* 3: 182-187.

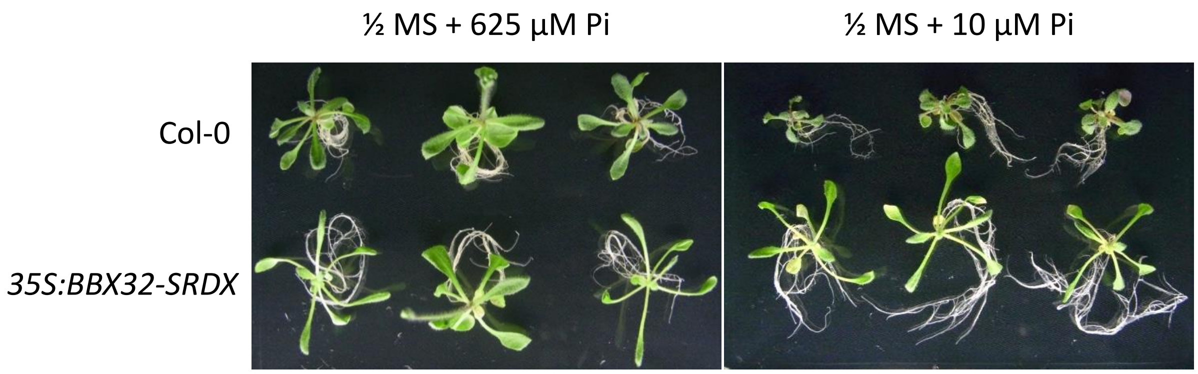

Supplementary Figure 1. The tolerance phenotype of *35S:BBX32-SRDX* plants to Pi deficiency. The wildtype (Col-0) and *35S:BBX32-SRDX* (line#2, 5, 7, 12) seedlings were grown on 1/2 MS medium in the presence of 625 or 10 μM Pi for 16 days.

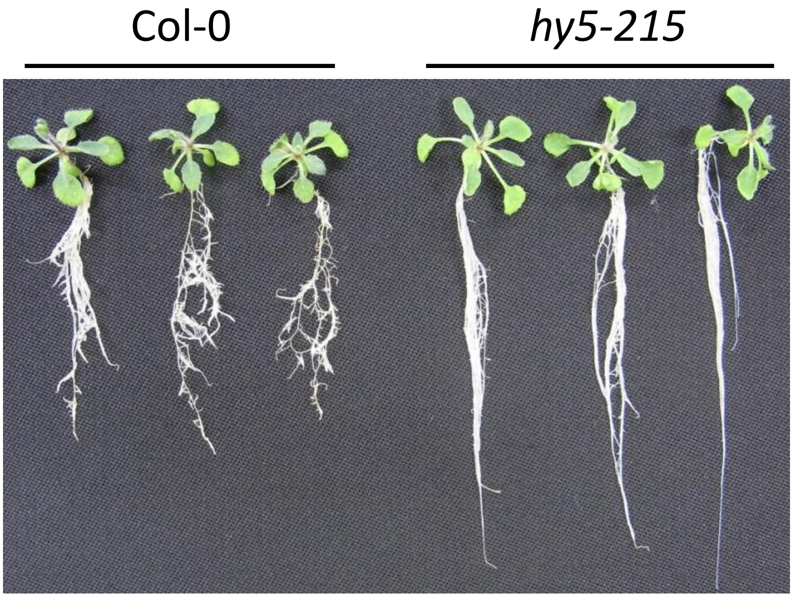

Supplementary Figure 2. The tolerance phenotype of mature *hy5-215* plants to Pi deficiency. The Col-0 and *hy5-215* seedlings were grown on 1/2 MS medium in the presence of 625 μM Pi for 12 days and then transferred to Pi-deficient condition for another 10 days.

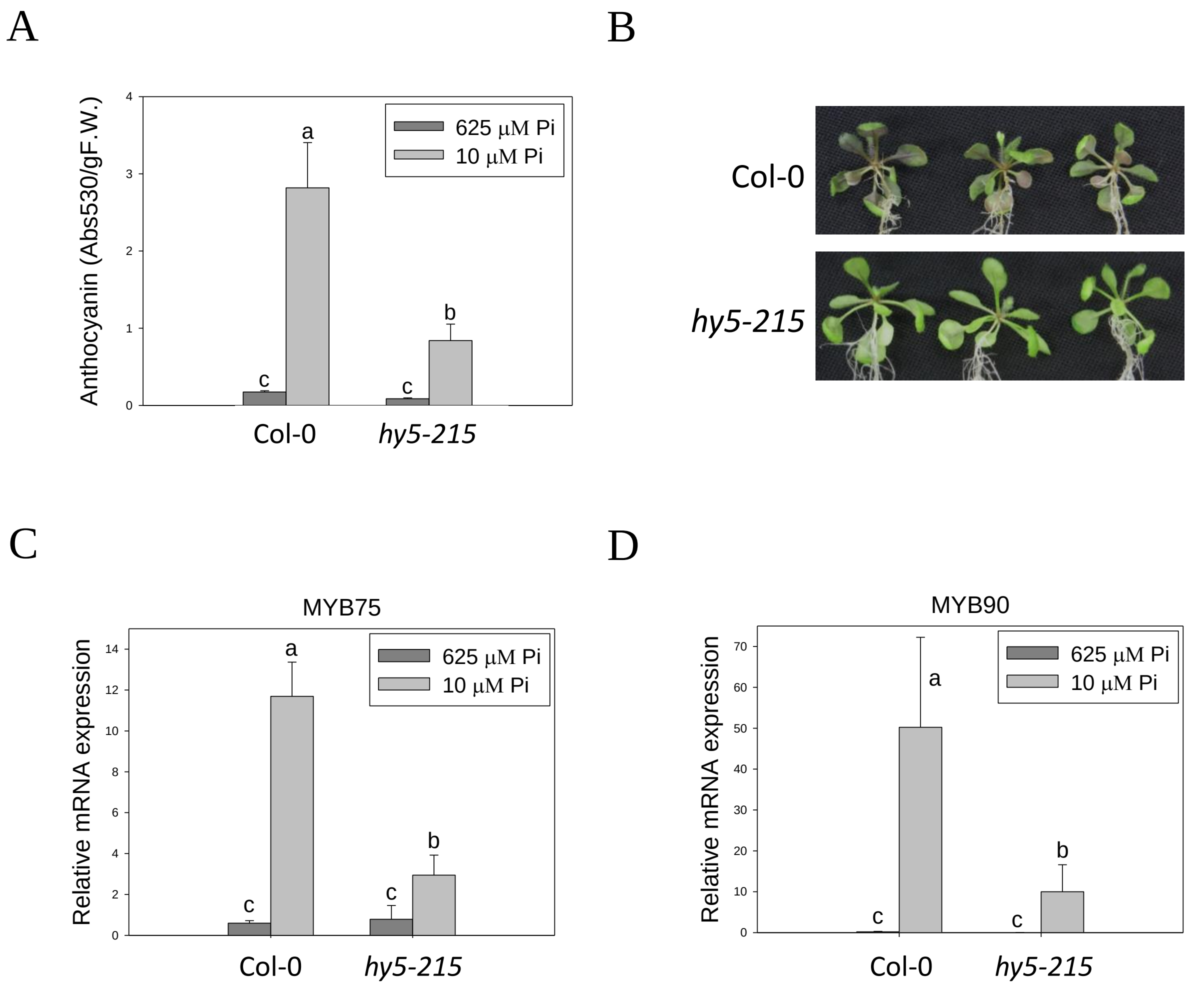

Supplementary Figure 3. Lower anthocyanin level in *hy5-215* mutants. Low Pi-induced anthocyanin accumulation in Col-0 and *hy5-215* (A-B). Anthocyanin content of 10-day-old seedlings was extracted by 45% methanol and 5% acetic acid. The relative level of anthocyanin was calculated from the absorbance at 530 and 637 nm. Expression level of TF genes involved in anthocyanin biosynthesis (C-D). RNA extracted from 10-day-old seedlings was subjected to real-time RT-PCR. Data represent means ± standard error (SE) of three independent experiments. Different letters above the bars indicate statistically significant differences among the means based on ANOVA followed by Fisher’s LSD test (P <0.05).

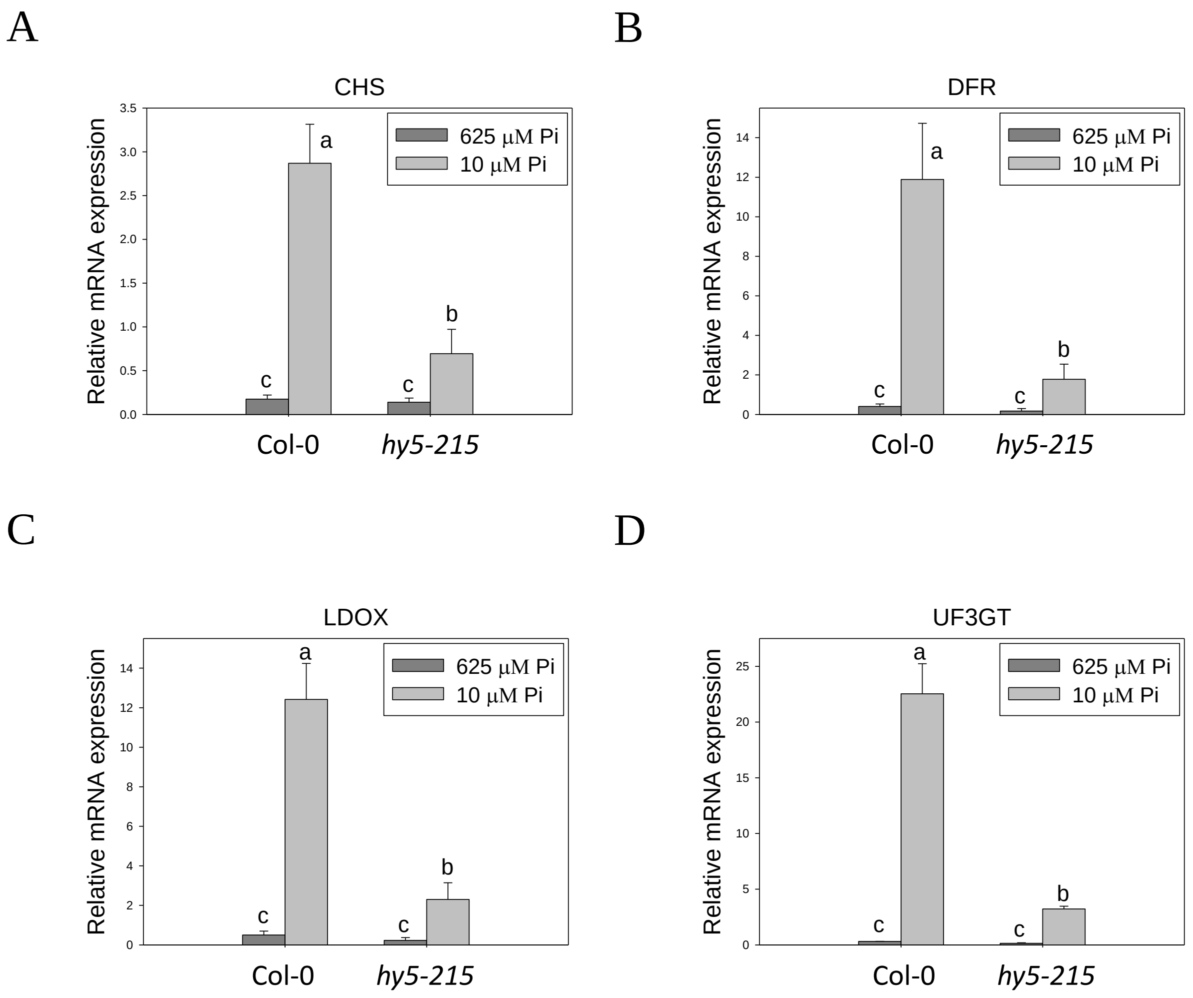

Supplementary Figure 4. Reduced expression of anthocyanin biosynthetic genes in *hy5-215* mutants. Expression of *CHS* (A), *DFR* (B), *LDOX* (C), and *UF3GT* (D) involved in anthocyanin biosynthesis were analyzed. RNA extracted from 10-day-old seedlings was subjected to real-time RT-PCR. Data represent means ± SE of three independent experiments. Different letters above the bars indicate statistically significant differences among the means based on ANOVA followed by Fisher's LSD test (P <0.05).

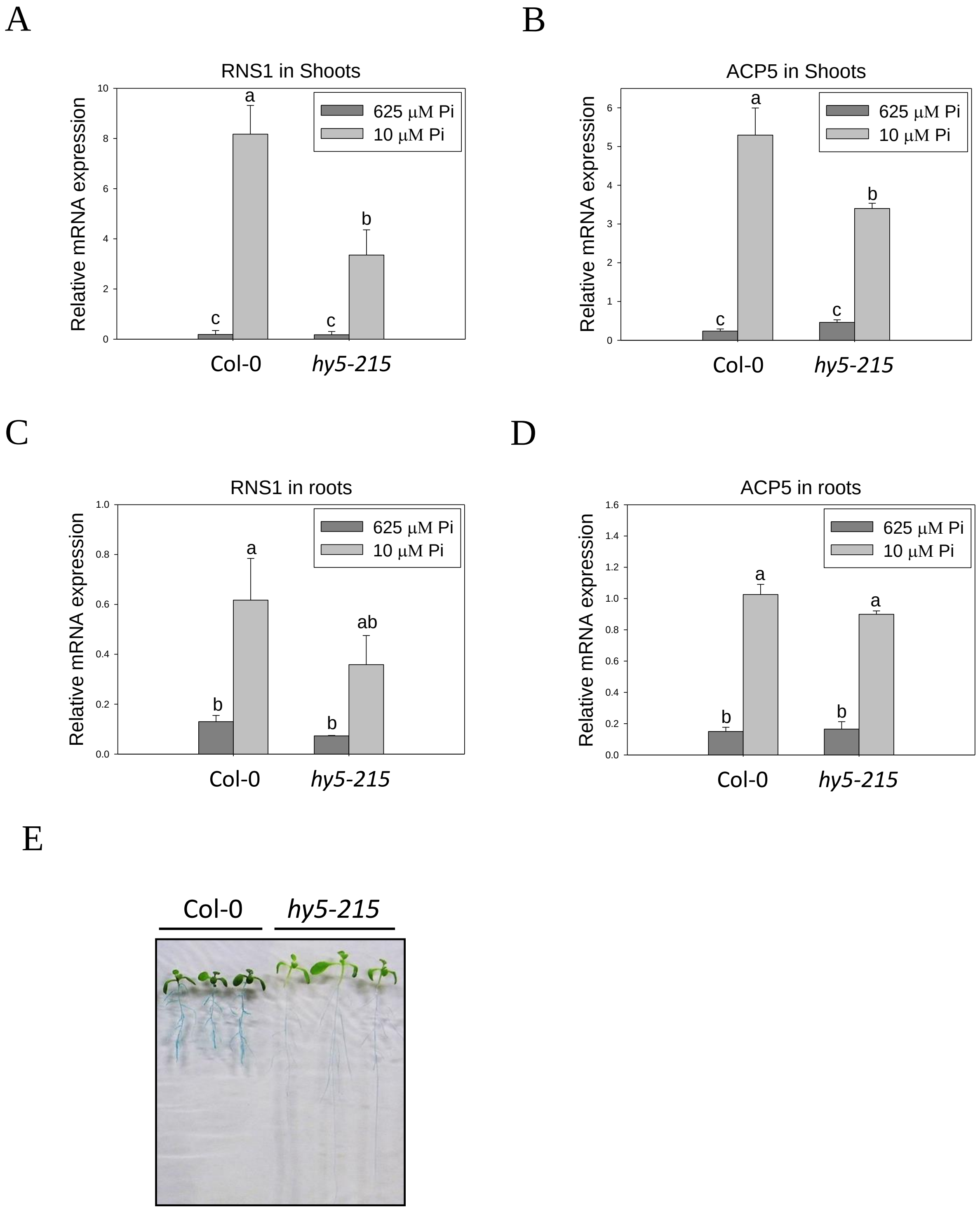

Supplementary Figure 5. Reduction of Pi deficiency-induced ribonucleases and acid phosphatases in *hy5-215* mutants. Expression of *RNS1* in shoots and roots (A,C). Expression of *ACP5* in shoots and roots (B,D). Acid phosphatase activity in response to Pi deficiency (E). The seedlings were grown on 1/2 MS medium with 625 or 10 μM Pi for 10 days. Total RNA of shoots and roots was extracted for real-time RT-PCR. For acid phosphatase activity, roots were overlaid with 0.01% BCIP, an acid phosphatase substrate, for 5 hours. The clear blue color in

Col-0 roots indicates higher acid phosphatase activity. Data represent means ± SE of three independent experiments. Different letters above the bars indicate statistically significant differences among the means based on ANOVA followed by Fisher's LSD test (P <0.05).

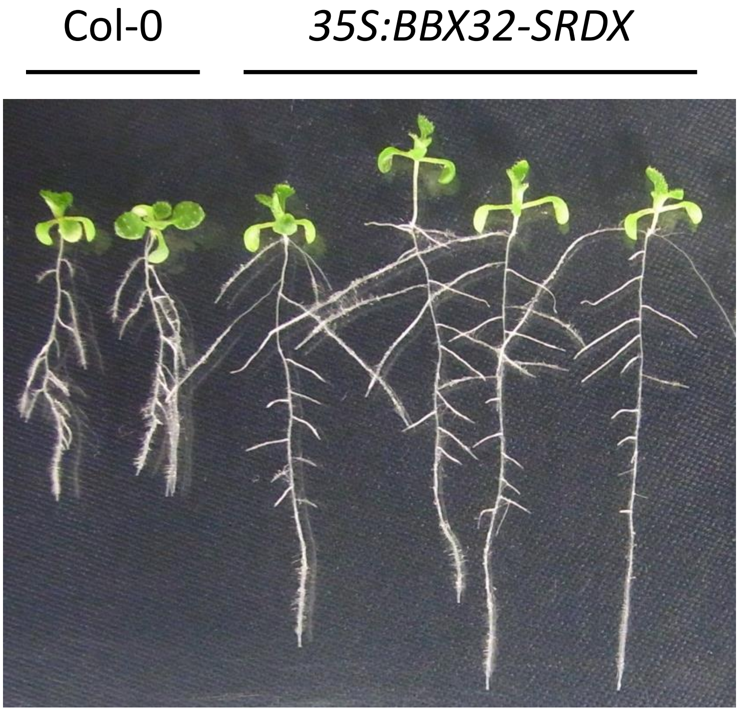

Supplementary Figure 6. Root architecture of wild-type and *35S:BBX32-SRDX* seedlings in response to Pi treatment. The seedlings were grown on 1/2 MS medium with 625 or 10 μM Pi and root architecture of Col-0 and *35S:BBX32-SRDX* was observed after growth of 10 days.

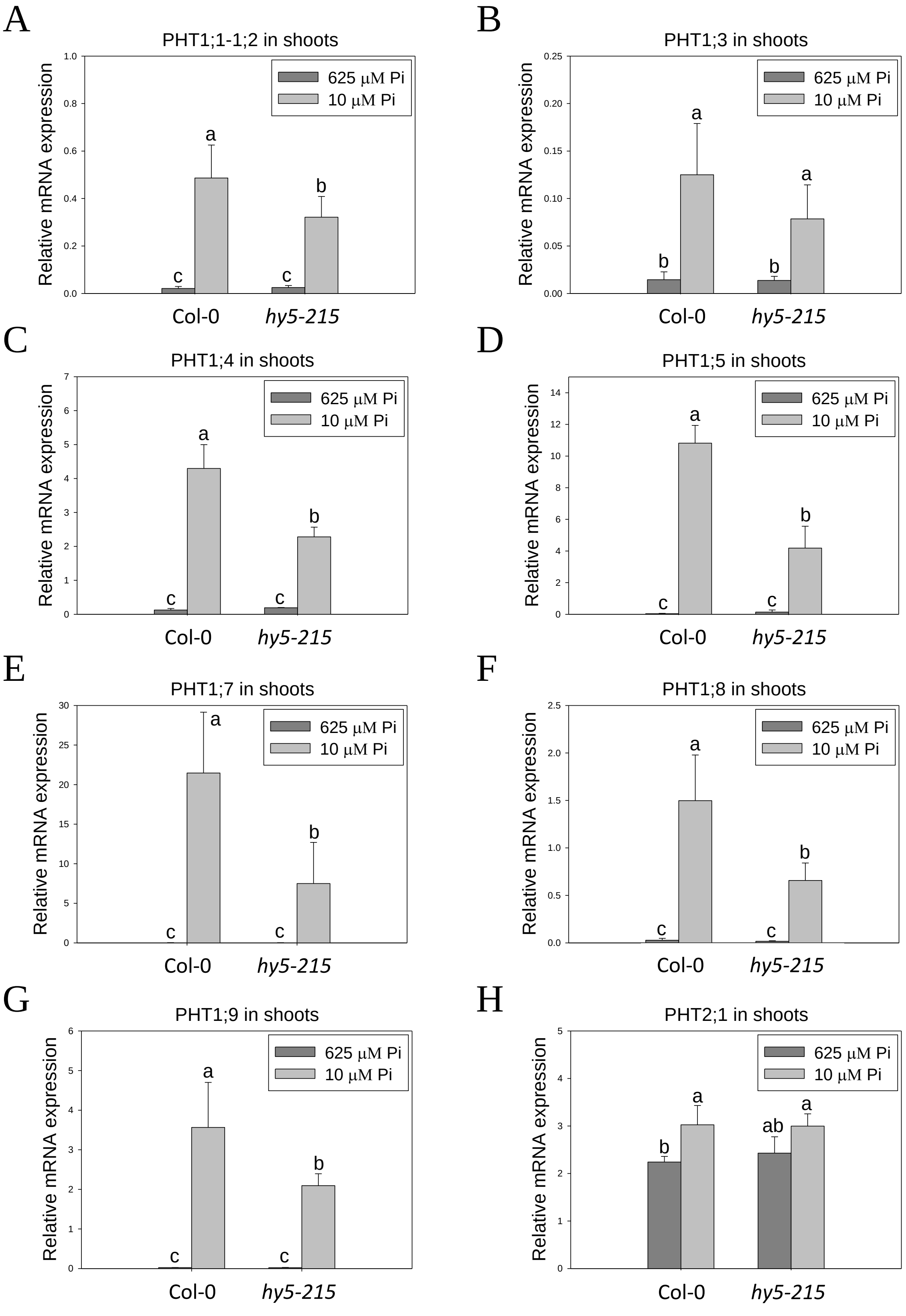

Supplementary Figure 7. Lower expression of phosphate transporter genes in *hy5-215* shoots. Expression levels of high-affinity Pi transporter family, *PHT1*, genes (A-G). Similar

expression pattern of low-affinity Pi transporter gene, *PHT2;1*, between Col-0 and *hy5-215* (H). RNA extracted from 10-day-old seedlings was subjected to real-time RT-PCR. Data represent means ± SE of three independent experiments.

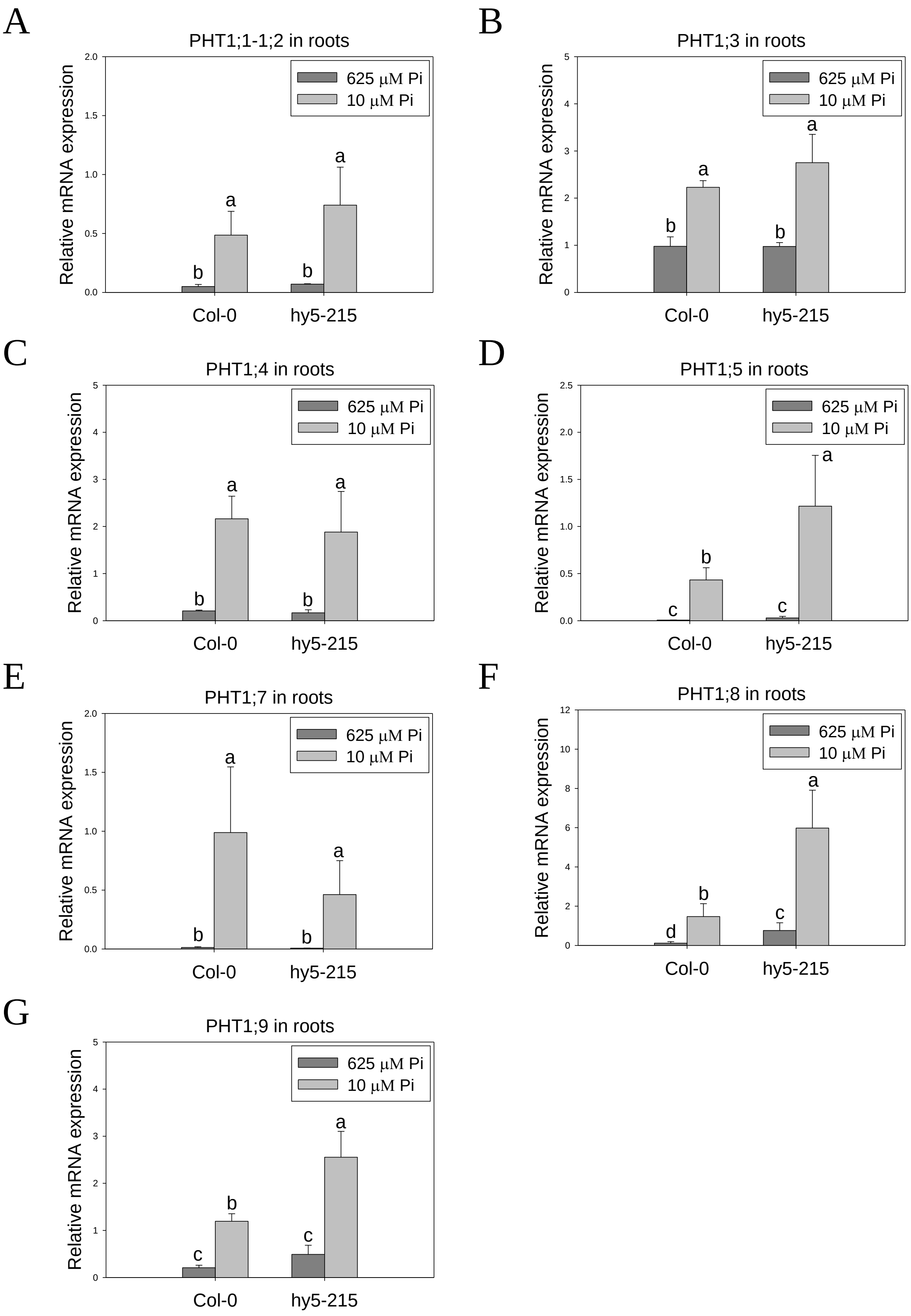

Supplementary Figure 8. Expression levels of phosphate transporter genes in *hy5-215* roots. Expression levels of high-affinity Pi transporter family, *PHT1*, genes (A-G). RNA

extracted from 10-day-old seedlings was subjected to real-time RT-PCR. Data represent means ± SE of three independent experiments.

A

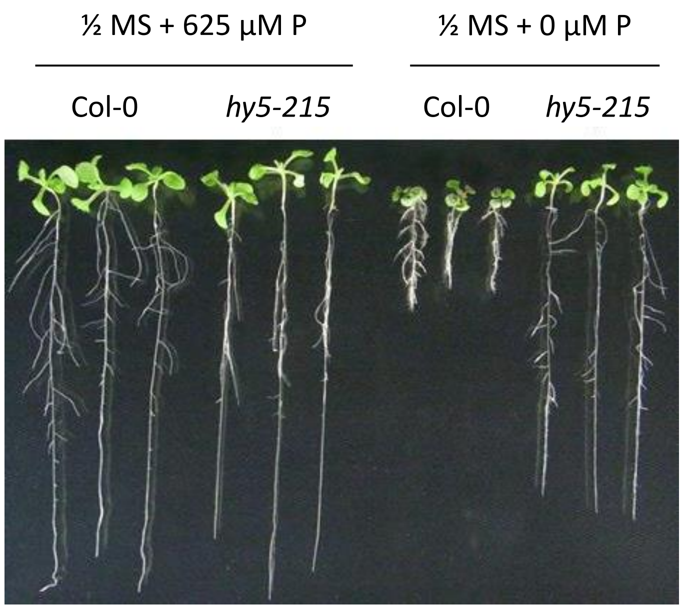

B

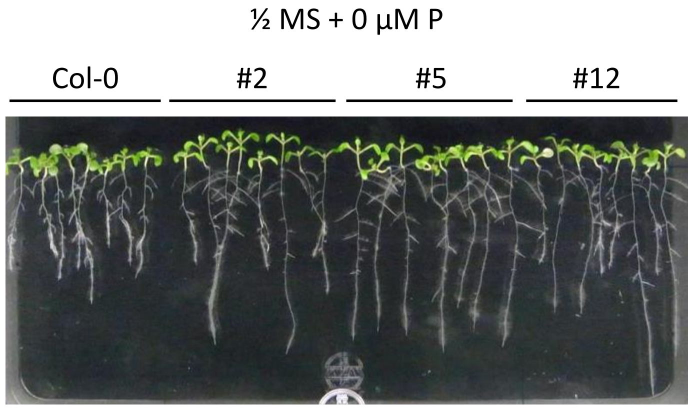

Supplementary Figure 9. Phenotype of *hy5-215* and *35S:BBX32-SRDX* seedlings grown in Pi-free condition. (A) Wildtype and *hy5-215* seedlings grown on Pi-sufficient and Pi-free media for 10 days. (B) Wildtype and *35S:BBX32-SRDX* (line#2, 5, 12) seedlings grown on Pi-free medium for 10 days.

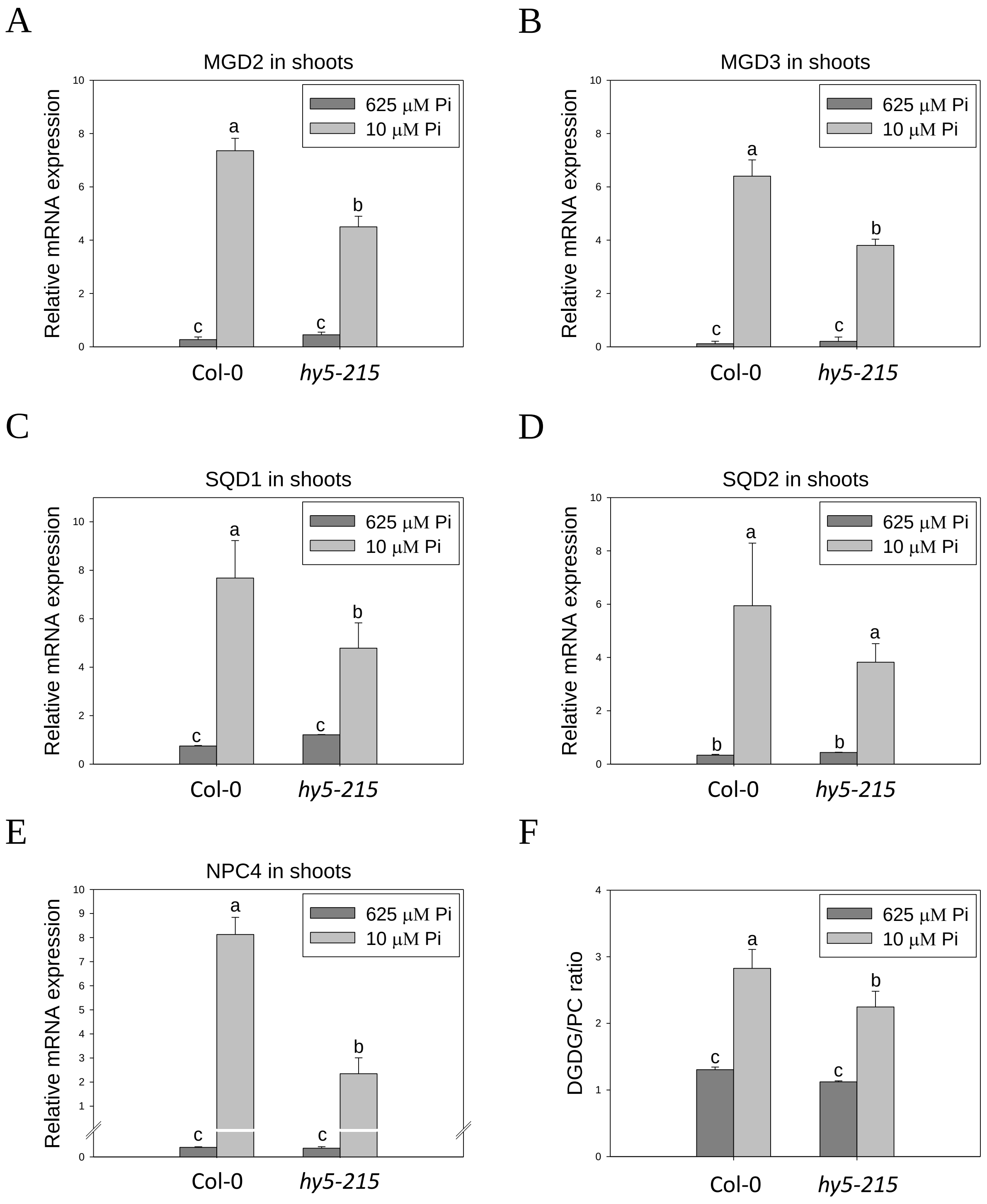

Supplementary Figure 10. Reduction of lipid remodeling in *hy5-215* mutants. Expression of *MGD2* (A), *MGD3* (B), *SQD1* (C) and *SQD2* (D) involved in glycolipid biosynthesis and *NPC4* (E) involved in phospholipid hydrolysis were analyzed. RNA extracted from 10-days-old seedlings was subjected to perform real-time RT-PCR. The DGDG/PC molar ratio in response to Pi deficiency (F). The seedlings were grown on 1/2 MS media with 625 or 10 μM Pi for 14 days. The leaves were harvested for analysis of membrane lipid composition by gas

chromatography. Data represent means ± SE of three independent experiments. Different letters above the bars indicate statistically significant differences among the means based on ANOVA followed by Fisher's LSD test (P <0.05).

A

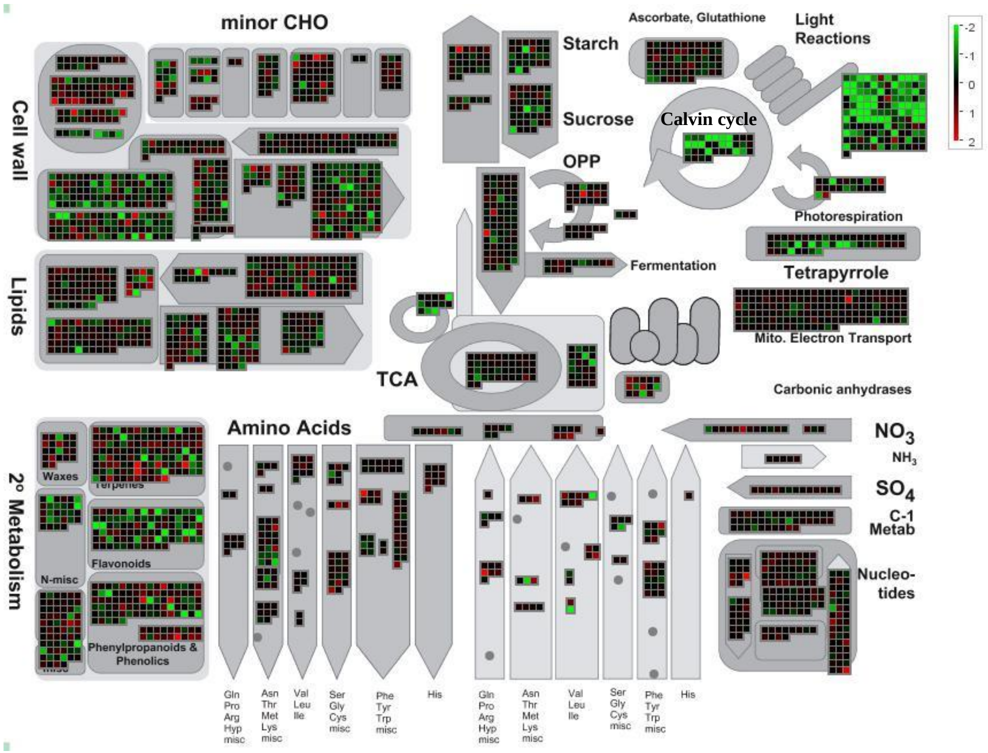

B

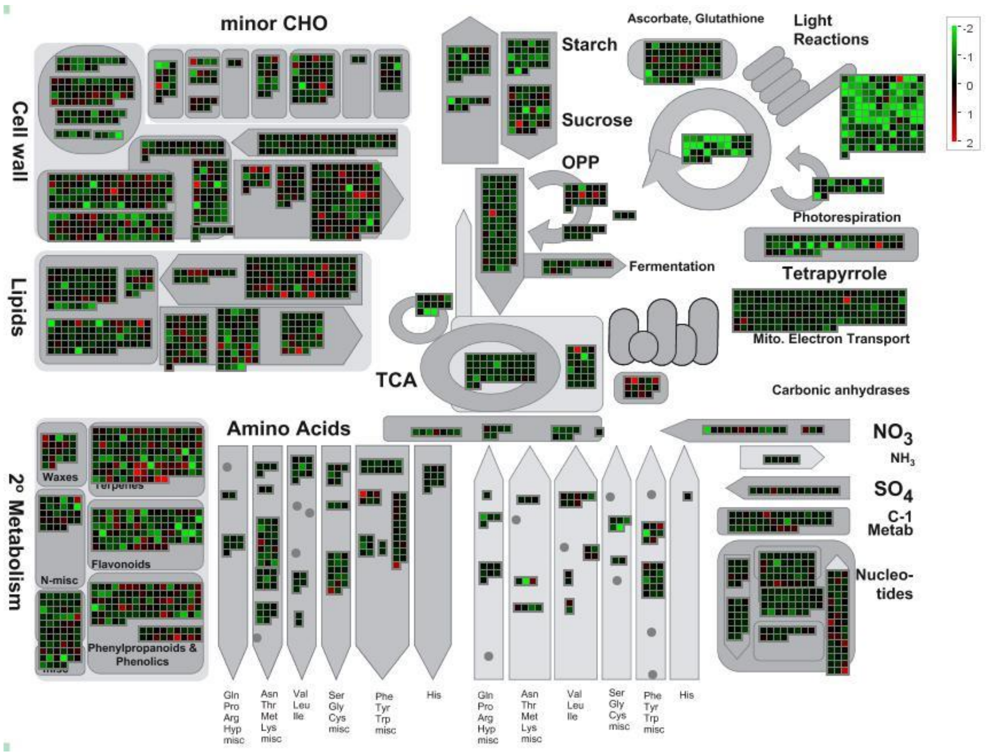

Supplementary Figure 11. Transcriptome analysis of *hy5-215* and *35S:BBX32-SRDX* in response to Pi deficiency by MapMan software. The MapMan metabolism overview maps

indicate a significant down-regulation of genes involved in photosynthesis in *hy5-215* (A) and *35S:BBX32-SRDX* (B) roots under Pi-deficient conditions. Each square in the graph represents one gene involved in different metabolisms. The expression levels (*hy5-215*/WT and *35S:BBX32-SRDX*/WT) calculated by $\log_2$ ratios were from three independent experiments.

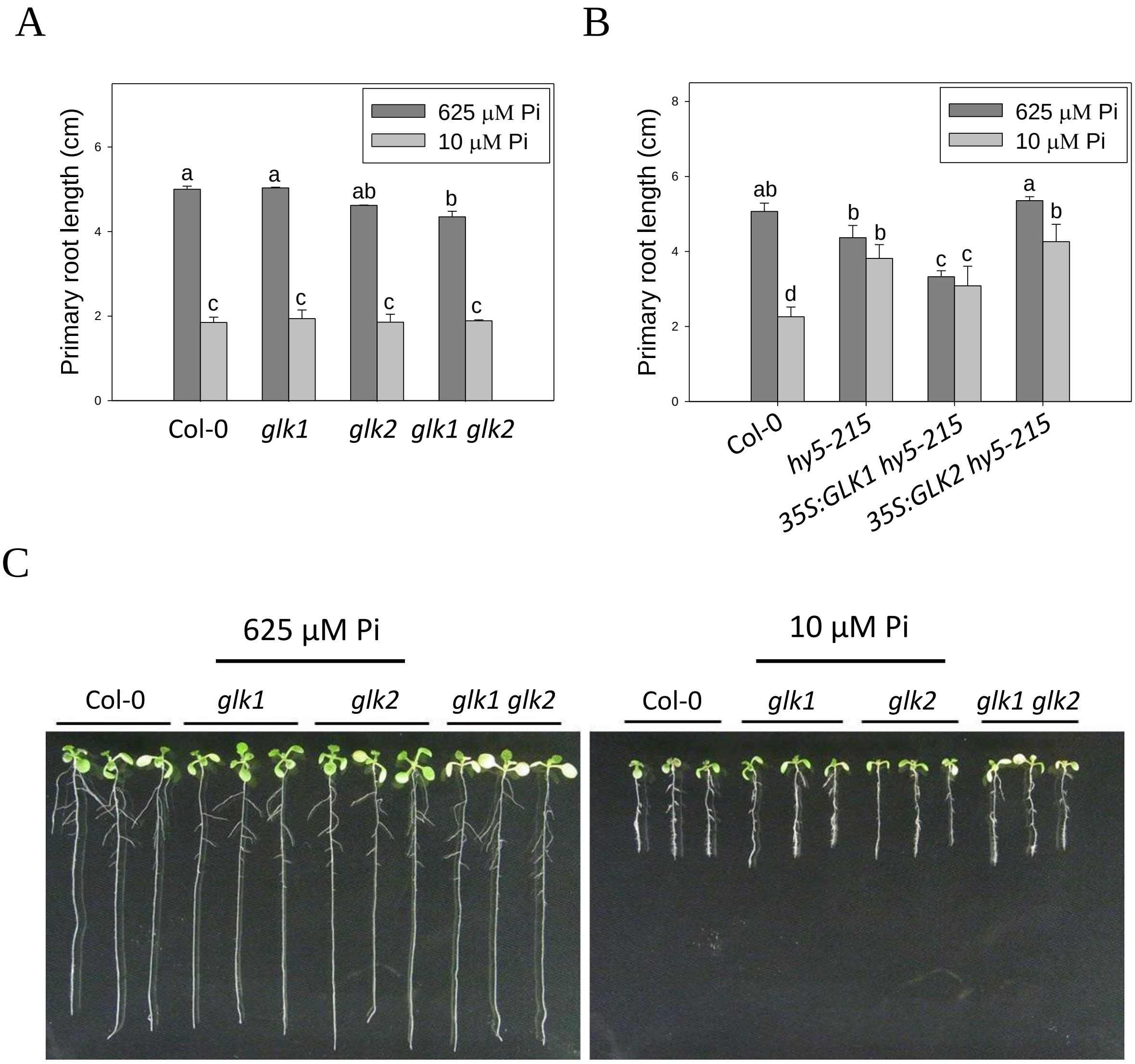

Supplementary Figure 12. Root growth of Arabidopsis *glk* mutants and *GLK* overexpression lines in response to Pi starvation. The PR lengths of *glk1*, *glk2* and *glk1 glk2* mutants (A,C). The PR lengths of 35S:*GLK1 hy5-215* and 35S:*GLK2 hy5-215* (B). The seedlings were grown on 1/2 MS media in the presence of 625 or 10 µM Pi. The PR length was measured after 10 days of growth. Two independent lines of 35S:*GLK1 hy5-215* and 35S:*GLK2 hy5-215* were used for the measurement of PR length. The average PR lengths are shown in the figure. Data represent means ± SE of four independent experiments. Different letters above the bars indicate statistically significant differences among the means based on ANOVA (Analysis of Variance) followed by Fisher's LSD (Least Significant Difference) test (P <0.05).

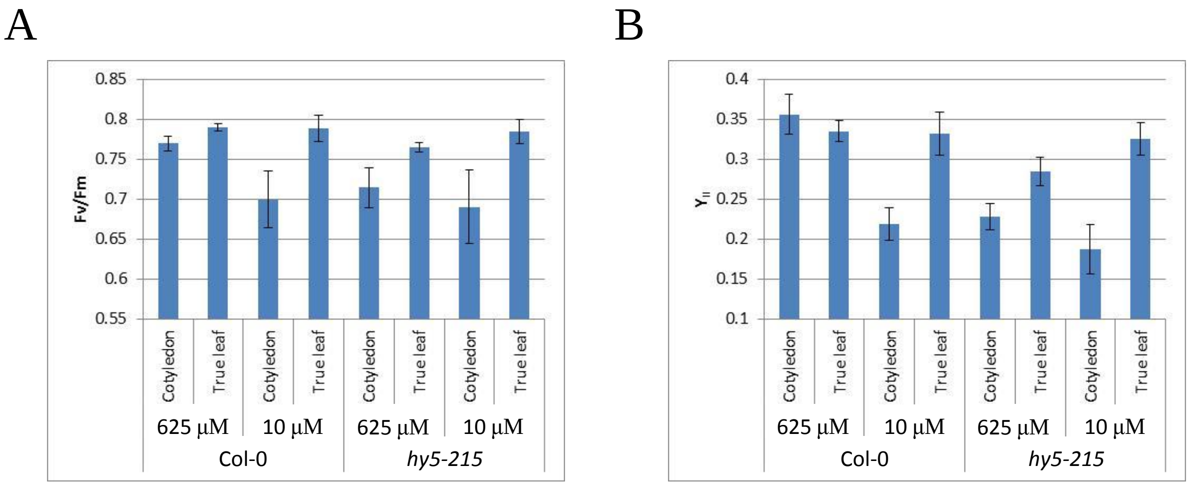

Supplementary Figure 13. Comparison of photosynthetic ability of *hy5-215* and wild type plants. (A) Measurement of maximum quantum yield of photosystem II (Fv/Fm). (B) Measurement of actual quantum yield of photosystem II in light (Y_{II}). The seedlings were grown on 1/2 MS media in the presence of 625 or 10 μM Pi. The Fv/Fm and Y_{II} were measured after 10 days of growth.

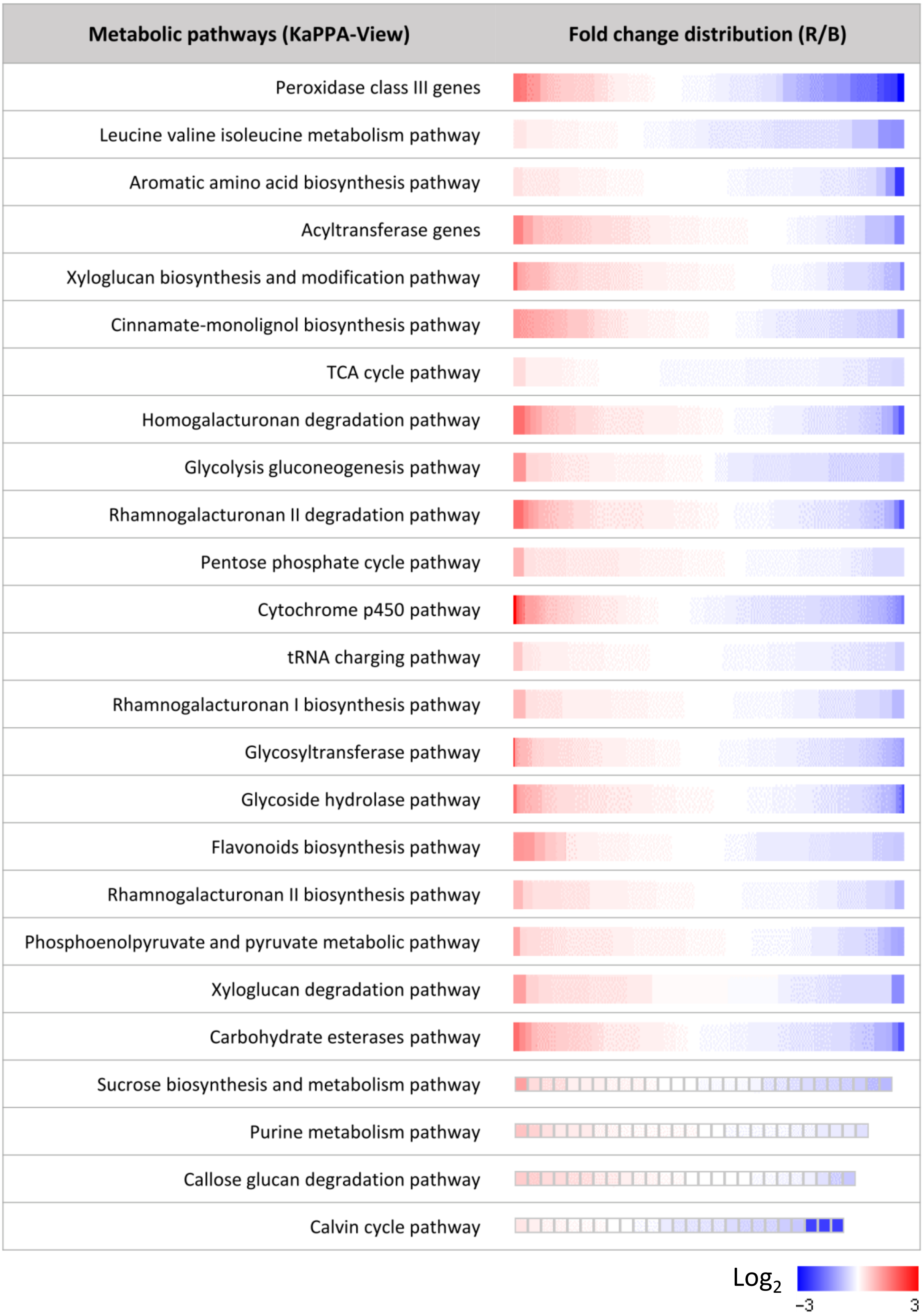
Metabolic pathways (KaPPA-View)
Fold change distribution (R/B)
Peroxidase class III genes
Leucine valine isoleucine metabolism pathway
Aromatic amino acid biosynthesis pathway
Acyltransferase genes
Xyloglucan biosynthesis and modification pathway
Cinnamate-monolignol biosynthesis pathway
TCA cycle pathway
Homogalacturonan degradation pathway
Glycolysis gluconeogenesis pathway
Rhamnogalacturonan II degradation pathway
Pentose phosphate cycle pathway
Cytochrome p450 pathway
tRNA charging pathway
Rhamnogalacturonan I biosynthesis pathway
Glycosyltransferase pathway
Glycoside hydrolase pathway
Flavonoids biosynthesis pathway
Rhamnogalacturonan II biosynthesis pathway
Phosphoenolpyruvate and pyruvate metabolic pathway
Xyloglucan degradation pathway
Carbohydrate esterases pathway
Sucrose biosynthesis and metabolism pathway
Purine metabolism pathway
Callose glucan degradation pathway
Calvin cycle pathway
Log2
−3
3

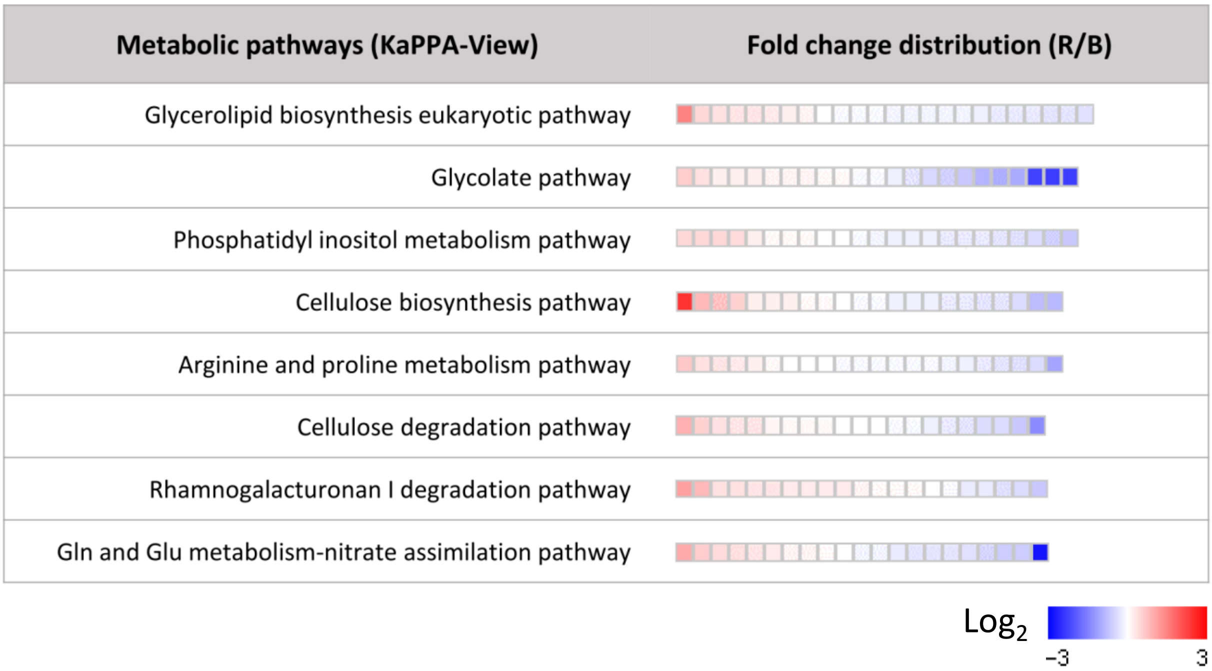

Supplementary Figure 14. Metabolic pathways affected by light quality under Pi-deficient condition. Effect of light quality on expression of genes encoding enzymes involved in different metabolic pathways in Arabidopsis roots was analyzed. The log2 fold change (R/B) was calculated by dividing the expression levels under R light treatment by the levels under B light treatment. The pathways with upregulated or downregulated genes in R light condition are presented.
